# Regulatory Functions of microRNAs in Cancer Stem Cells: Mechanism, Facts, and Perspectives

**DOI:** 10.3390/cells14141073

**Published:** 2025-07-14

**Authors:** Xingmei Mao, Sixue Peng, Yan Lu, Linjiang Song

**Affiliations:** School of Medical and Life Sciences, Chengdu University of Traditional Chinese Medicine, Chengdu 611137, China; mxm18328161362@163.com (X.M.); pengsixuezora@163.com (S.P.); luyan1275@163.com (Y.L.)

**Keywords:** miRNAs, cancer stem cells, chemotherapy resistance, biomarkers, signaling pathways

## Abstract

Cancer represents a significant global health hazard marked by elevated morbidity and mortality rates. Furthermore, the majority of tumor therapies encounter challenges, including metastasis, recurrence, and drug resistance. Consequently, it is essential to identify a specific and efficient tumor treatment approach. In recent years, the ongoing investigation and comprehension of tumors have led to significant attention towards cancer stem cells (CSCs). CSCs can facilitate tumor progression via self-renewal, differentiation capabilities, and multidrug resistance. Their function as a fundamental contributor to tumor heterogeneity, drug resistance, recurrence, and metastasis has emerged as a significant focus in cancer therapy research. In recent years, microRNAs (miRNAs) have been identified as crucial post-transcriptional regulators in biological processes, including chemosensitivity, self-renewal, apoptosis, invasion, and metastasis of cancer stem cells (CSCs). This paper systematically reviews the molecular mechanisms through which miRNAs influence the characteristics of cancer stem cells by targeting essential genes (e.g., SOX2, *EGFR*, c-Met) and modulating signaling pathways, including Wnt/β-catenin, Notch, Hedgehog, and PI3K/Akt. Furthermore, we investigated the viability of miRNAs as non-invasive biomarkers for cancer diagnosis and prognosis evaluation, examined the similarities and attributes of pivotal miRNAs in modulating cancer stem cell functionality, and deliberated on therapeutic approaches stemming from miRNA regulation of cancer stem cell activity. We anticipate that this research will yield novel insights into targeted cancer therapy.

## 1. Introduction

Cancer has long been at the top of the list of diseases that are a significant threat to human health globally, both in terms of incidence and mortality rates. The most recent GLOBOCAN figures show that there were 19.96 million new cases of cancer worldwide in 2022, along with roughly 9.73 million fatalities. Breast cancer and lung cancer are the most frequent cancers in women and men, respectively. Lung cancer (14.1%), breast cancer (11.7%), and colorectal cancer (9.3%) were among the top three in terms of incidence rates [1,2]. Drug resistance, metastasis, and tumor recurrence remain significant therapeutic challenges despite improvements in conventional therapies, including radiation, chemotherapy, and surgery [2]. In recent years, the theory of cancer stem cells (CSCs) has provided a new perspective on the biological properties of cancer.

Cancer stem cells are a distinct subset of cells in tumor tissues with self-renewal, multidirectional differentiation potential, and strong tumorigenic activity. The American Association for Cancer Research (AACR) defines cancer stem cells (CSCs) as “cells within a tumor that possess self-renewal capabilities and can generate a diverse lineage of cancer cells” [3]. The investigation of cancer stem cells (CSCs) commenced in 1994, when John Dick’s team successfully isolated the CD34^+^ CD38^−^ CSC subpopulation in acute myeloid leukemia (AML) and identified a CD38^−^ CSC subpopulation in AML, thereby establishing a tumor hierarchy model that provided a foundational framework for CSC research [4]. From 2003 to 2006, Michael Clarke’s team [5] and Peter Dirks’ team [6] validated the presence of cancer stem cells (CSCs) in solid tumors, a groundbreaking revelation that challenged the conventional notion of tumor cell homogeneity and facilitated the advancement of the tumor cell homogeneity concept. This groundbreaking discovery fundamentally challenged the conventional perspective of “tumor cell homogeneity” and led to the widespread acknowledgment of cancer stem cells (CSCs).

Research indicates that CSCs comprise about 0.1–2% of the overall tumor cell population; yet, they play a crucial role in tumor heterogeneity, facilitating drug resistance, and contributing to recurrence and metastasis [7,8]. CSCs preserve their stemness attributes by activating critical signaling pathways, including Wnt/β-catenin, Notch, and Hedgehog while expressing distinct surface markers such as CD44, CD133, and ALDH1 [3,9,10]. In recent years, studies on CSCs have revealed their interactions in the tumor microenvironment and mechanisms of response to therapy. By targeting specific markers of CSCs or modulating their key signaling pathways, it is expected that more effective cancer therapeutic strategies can be developed to fundamentally inhibit tumor recurrence and metastasis and improve the prognosis of cancer patients.

As research advances, the emphasis on cancer stem cells (CSCs) has transitioned from the question of their “existence” to a comprehensive understanding of their fundamental roles (e.g., facilitating tumor malignancy), molecular mechanisms (encompassing signaling, epigenetic regulation, and metabolic reprogramming), state plasticity [11], microenvironmental reliance, and interactions with the immune system [12]. The theory offers a fundamental foundation for clarifying obstinate characteristics of cancers, including recurrence, metastasis, and medication resistance. Investigating the molecular mechanisms of cancer stem cells (CSCs) is anticipated to facilitate the development of more efficient therapeutic options to limit tumor recurrence and metastasis at their source, ultimately enhancing patient prognosis.

The elucidation of the molecular regulatory network of cancer stem cells (CSCs) is a pivotal advancement in addressing treatment resistance, as it serves as the fundamental mechanism propelling malignant tumor progression. In recent years, the significance of epigenetic regulatory mechanisms, particularly non-coding RNAs, in preserving cancer stem cell stemness has gained prominence. MicroRNAs have emerged as essential molecular switches for elucidating the biological activity of cancer stem cells due to their synergistic control of several targets [13,14]. MicroRNAs (miRNAs) are a class of small non-coding RNAs, approximately 20–22 nucleotides long, that modulate gene expression at the post-transcriptional level by binding to the 3′ untranslated region (3′ UTR) of target mRNA, thus inhibiting its translation or promoting its degradation. miRNAs play an essential role in numerous biological processes, including cell proliferation, differentiation, apoptosis, metabolism, and embryonic development. Nearly sixty percent of protein-coding genes in the human genome are modulated by miRNAs, and their dysregulated expression is significantly associated with tumorigenesis [7]. These molecules participate in essential biological processes like cell proliferation, differentiation, apoptosis, and metabolism. It is believed that around 60% of human protein-coding genes are regulated by miRNAs, and their dysregulated expression is intimately associated with cancer and development. Significantly, miRNA activity is meticulously regulated. Competing endogenous RNAs (ceRNAs) (e.g., circRNA, lncRNA) are a category of non-coding RNAs that can interact with miRNAs and possess miRNA response elements (MREs) that are complementary to miRNAs. CeRNAs can competitively bind to miRNAs, diminishing the likelihood of miRNA binding to target mRNAs, which indirectly alleviates the inhibitory impact of miRNAs on target mRNAs and restores or enhances their expression levels [15].

In cancer, miRNAs exhibit aberrant expression, with specific miRNAs functioning as tumor suppressors or oncogenes, influencing tumor cell proliferation, invasion, metastasis, and drug resistance through the regulation of oncogenes or tumor suppressor genes. A multitude of studies has demonstrated that particular miRNAs play a significant role in CSC self-renewal, multidirectional differentiation, metabolic reprogramming, and immune evasion by directly modulating core CSC pathways, including Wnt/β-catenin, Notch, and Hedgehog, or by regulating networks of stemness transcription factors such as OCT4, SOX2, and NANOG [14,16]. This multilayer regulatory network’s capacity to bi-directionally influence the fate of cancer stem cells offers a novel approach for targeting their elimination. The consistent presence of miRNAs in bodily fluids and the significant association between their expression profiles and disease states render them valuable as non-invasive diagnostic indicators and therapeutic targets.

Currently, research focusing on the miRNA-CSC regulatory network have emerged as an important field of interest in cancer therapy. Investigating the molecular mechanisms of miRNAs in CSCs facilitates the development of miRNA-based biomarkers and offers a theoretical foundation for creating innovative therapeutic strategies targeting CSCs [2,17].

## 2. The Function of miRNAs in Modulating the Chemosensitivity and Radiosensitivity of CSCs

**Figure 1:**
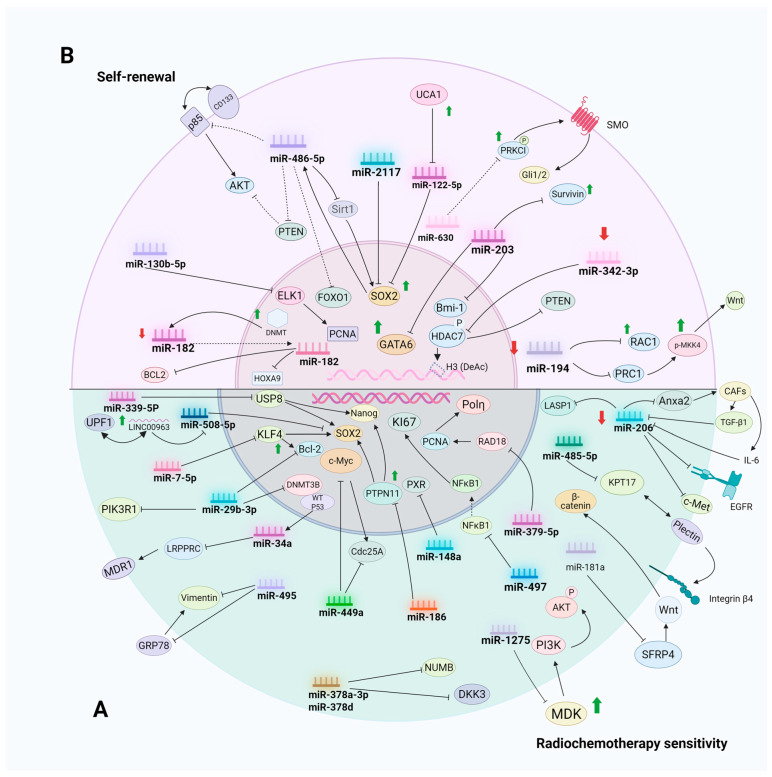
Molecular mechanisms of miRNAs regulating radiochemotherapy resistance and self-renewal in CSCs. (**A**) Molecular mechanisms of miRNAs regulating chemoresistance and radioresistance in CSCs. miR-339-5p, miR-508-5p, miR-7-5p, miR-29b-3p, miR-449a, and miR-186 downregulation promotes the expression of oncogenes such as SOX2, Bcl-2, c-Myc, and Nanog, which in turn leads to chemoresistance and radiotherapy resistance. miR-206 downregulation in multiple cancer cells promoted chemoresistance through activation of the Wnt/β-catenin signaling pathway; miR-181a activated this pathway through inhibition of SFRP4 and enhanced chemoresistance in CSCs. *p53* mutations caused chemoresistance through the miR-34a/LRPPRC/MDR1 signaling pathway. Downregulation of miR-497 promoted gemcitabine resistance in CSCs by enhancing NFκB1 activity. Overexpression of miR-379-5p inhibited radiotherapy resistance in CSCs by regulating the RAD18/Polη axis. miR-495 downregulation promoted the EMT phenotype through upregulation of GRP78, resulting in radiotherapy resistance. miR-485-5p deregulation enhanced chemoresistance by modulating the KRT17/integrin/FAK/Src/ERK/β-catenin signaling pathway to enhance chemoresistance. Overexpression of miR-1275 inhibits the MDK/AKT signaling pathway and attenuates chemoresistance. miR-378a-3p/miR-378d promotes chemoresistance in CSCs through inhibition of DKK3 and NUMB. miR-148a deregulation enhances chemoresistance in CSCs through upregulation of PXR. (**B**) Molecular mechanisms by which miRNAs regulate self-renewal of CSCs. miR-2117 downregulation increases SOX2 expression. miR-194 attenuates the regulation of RAC1 and PRC1 and activates the Wnt/β-catenin signaling pathway. miR-342-3p deregulation enhances CSCs by inhibiting PTEN and promoting HDAC7 self-renewal. miR-130b-5p was negatively correlated with ELK1, which is associated with self-renewal of CSCs. Downregulation of miR-203 promoted the expression of self-renewal-associated factors GATA6, survivin, and Bmi-1. miR-630 attenuated the inhibition of PRKCI, leading to the activation of the Hedgehog signaling pathway. miR-486-5p was downregulated, activating the p85/AKT pathway, Fox01, Sirt1 expression, and enhanced CSC self-renewal. lncRNA UCA1 regulates CSC self-renewal by inhibiting the microRNA-122-5p/SOX2 axis. miR-182 silencing induced by DNA hypermethylation targets BCL2 and HOXA9 and promotes CSC self-renewal.

Despite significant advances in cancer treatment over the past decades, resistance to chemotherapy and radiotherapy remains a significant problem in cancer treatment. It is estimated that 90% of deaths in cancer patients are related to drug resistance [18]. CSCs are a subpopulation of tumor cells with self-renewal, multidirectional differentiation potential, and drug resistance. They are highly resistant to chemotherapy and radiotherapy and are considered to be the main source of tumor recurrence and metastasis [19,20]. Recent studies indicate that miRNAs are pivotal in modulating drug resistance in CSCs. By targeting particular genes or signaling pathways, CSCs can modulate essential biological processes such as self-renewal, proliferation, and apoptosis, consequently influencing their responsiveness to chemotherapeutic agents and radiotherapy. Table 1 and Figure 1A demonstrate the molecular mechanisms by which multiple miRNAs regulate chemoresistance and radiotherapy resistance in CSCs.

### 2.1. Regulatory Function of miRNAs in the Chemosensitivity of CSCs

#### 2.1.1. miR-378a-3p and miR-378d

Exosomes miR-378a-3p and miR-378d enhance the stem cell characteristics of breast cancer stem cells by modulating the WNT/β-catenin pathway and Notch signaling.

Study shows that chemotherapeutic agents, such as doxorubicin and paclitaxel, activate the EZH2/STAT3 signaling pathway, resulting in heightened secretion of exosomes enriched with miR-378a-3p and miR-378d.Upon uptake by chemoresistant tumor cells, these exosomes activate the WNT/β-catenin and Notch signaling pathways by inhibiting the WNT pathway inhibitor DKK3 and the Notch pathway inhibitor NUMB, thereby augmenting the characteristics of the BCSCs and contributing to chemoresistance.

The study also found that the application of EZH2 inhibitors (e.g., tazemetostat) markedly diminished the concentrations of miR-378a-3p and miR-378d in exosomes, reinstated the expression of DKK3 and NUMB, and impeded the activation of pertinent signaling pathways, thus reversing chemoresistance [21].

#### 2.1.2. miR-1275

miR-1275 was markedly downregulated in the plasma and in chemotherapy-resistant BC cell lines (MCF-7/ADR) from patients with chemotherapy-resistant breast cancer. Reduced levels of miR-1275 were significantly correlated with diminished overall survival in patients.

Research demonstrated that the overexpression of miR-1275 markedly increased the sensitivity of breast cancer cells to chemotherapeutic agents, including epirubicin, by directly targeting the 3′UTR of *MDK* (mesonephrine). MDK is a secreted protein that activates the PI3K/AKT signaling pathway, thereby imparting chemoresistance to cells. miR-1275 suppressed the activation of the PI3K/AKT signaling pathway by targeting MDK, consequently diminishing the characteristics of BCSCs and improving chemosensitivity [22].

#### 2.1.3. miR-508-5p

In endometrial cancer (EC), miR-508-5p acts as a tumor suppressor. Reduced expression of miR-508-5p is associated with chemoresistance and the characteristics of ECSCs.

miR-508-5p was found to inhibit *SOX2* expression by targeting its 3′UTR, consequently diminishing the self-renewal capacity, chemoresistance, migration, and invasion of ECSCs. SOX2 is a fundamental transcription factor that governs development and the pluripotency of stem cells. In cancer, it serves as a principal oncogenic driver by preserving tumor stem cell characteristics, promoting epithelial–mesenchymal transition (EMT), activating oncogenic pathways, and fostering treatment resistance [39,40].

LINC00963 functions as a ceRNA, binding to miR-508-5p, thereby neutralizing its inhibitory impact on SOX2 and facilitating chemoresistance. Moreover, Up-frameshift mutant 1 (UPF1) is significantly expressed in ECSCs and may control the behavior and destiny of ECSCs by stabilizing LINC00963. The simultaneous inhibition of UPF1 and LINC00963 significantly diminished the tumorigenic potential of ECSCs.

In conclusion, the UPF1/LINC00963/miR-508-5p/SOX2 axis is significant for investigating chemotherapy resistance in endometrial cancer, potentially serving as a novel therapeutic target [23].

#### 2.1.4. miR-181a

In ovarian cancer, miR-181a promotes the Wnt/β-catenin signaling pathway by targeting and inhibiting SFRP4, a Wnt signaling pathway inhibitor, hence augmenting ovarian cancer stem cell (OCSC) traits and generating resistance to platinum-based chemotherapy.

Research indicates that elevated levels of miR-181a in ovarian cancer cells are significantly correlated with resistance to treatment. Decreased expression of SFRP4, a secreted Wnt inhibitor, is linked to unfavorable prognosis in ovarian cancer. The inhibitory influence of miR-181a on SFRP4 mitigates its negative modulation of the Wnt pathway, hence activating the Wnt/β-catenin signaling cascade, resulting in the accumulation of β-catenin protein and the overexpression of Wnt target genes (including ALDH, CD133, SOX2, CTNNB1, TCF7, LGR5, and LEF1) [24].

#### 2.1.5. miR-485-5p

In oral squamous cell carcinoma (OSCC), miR-485-5p markedly suppresses the self-renewal, proliferation, and epithelial–mesenchymal transition (EMT) of cancer stem cells (CSCs) by targeting keratin 17 *(KRT17*) and its associated signaling pathways, consequently diminishing CSC invasiveness and resistance to chemotherapy.

Research indicates that KRT17 expression is markedly increased in highly invasive OSCC cell lines and advanced-stage tumor samples, correlating with unfavorable patient prognoses. KRT17 mechanistically activates integrin β4/α6 by its binding to nidiferrin, which promotes the phosphorylation of FAK, Src, and ERK; stabilizes β-catenin nuclear transport; upregulates stemness markers, including CD44 and EGFR; and enhances the stemness characteristics and drug resistance of OSCC.

miR-485-5p directly suppresses KRT17 expression, diminishes tumor spheroid formation, and increases cellular sensitivity to cisplatin/carboplatin. Targeting KRT17 or employing the Src inhibitor dasatinib can suppress tumor growth and improve chemotherapy sensitivity by obstructing the integrin/β-catenin pathway [25].

#### 2.1.6. miR-148a

In CSCs, the overexpression of Pregnane X Receptor (PXR) protein significantly contributes to chemoresistance and tumor recurrence. PXR functions as a transcription factor that regulates the expression of genes linked to chemoresistance in CSCs. miR-148a can modulate the expression of PXR at the post-transcriptional level by sequence-specific hybridization with the 3′ UTR of PXR mRNA, demonstrating a negative correlation between the two.

Research demonstrated that in colorectal cancer stem cells, the expression of miR-148a was downregulated, facilitating the preferential expression of PXR. Overexpression of miR-148a can suppress the expression and activity of PXR and diminish the expression of PXR downstream target genes, including *ALDH1A1 and ABCG2*, thereby inhibiting the stem cell phenotype of CSCs and concurrently reducing their resistance to chemotherapy. Post-chemotherapy, miR-148a diminished the enrichment of CSCs and postponed tumor recurrence following chemotherapy.

Furthermore, it was discovered that the anti-helminthic agent clonidine could stimulate miR-148a expression, consequently suppressing PXR expression and diminishing the population of CSCs while also enhancing the efficacy of chemotherapeutic agents to mitigate CSC chemoresistance and tumor recurrence [26].

Moreover, numerous recent studies have elucidated the molecular mechanisms through which specific miRNAs govern drug resistance in cancer stem cells across various cancer types.

For instance, in hepatocellular carcinoma (HCC), miR−186 markedly enhanced the apoptosis rate of cisplatin-treated HCC cells by targeting *PTPN11* and elevating the expression of apoptosis-related proteins [27]. In non-small-cell lung cancer (NSCLC), miR-206 diminished chemoresistance by targeting *c-Met* [28]. In LCSCs, miR-206 impeded tumor progression and chemoresistance by targeting the epidermal growth factor receptor (*EGFR*) [29]. Additionally, in intrahepatic cholangiocarcinoma, miR-206 augmented the sensitivity to the chemotherapeutic agent gemcitabine by obstructing the interaction between cancer-associated fibroblasts (CAFs) and tumor cells [30].

In colorectal cancer, miR-34a suppresses the expression of multidrug resistance protein 1 (MDR1) and enhances chemosensitivity to 5-FU by targeting *LRPPRC*, while *P53* mutations foster chemoresistance by inhibiting miR-34a [31]. In pancreatic cancer, the overexpression of miR-497 markedly diminished the resistance of PCSCs to gemcitabine by targeting nuclear factor κB1 (*NFκB1*) [32]. In ovarian cancer, miR-379-5p inhibited the recruitment of mono-ubiquitylated PCNA to DNA polymerase η (Polη) by modulating DNA repair-associated genes (e.g., *RAD18*), leading to impaired DNA damage repair, which subsequently activated cell cycle arrest and apoptosis pathways, thereby increasing the susceptibility of cancer cells to the chemotherapeutic agent cisplatin [33].

The findings indicate that miRNAs significantly regulate drug resistance in cancer stem cells, and targeting specific miRNAs may serve as an effective strategy to surmount chemotherapy resistance in cancer.

### 2.2. The Regulatory Function of miRNAs in the Susceptibility of CSCs to Radiotherapy

#### 2.2.1. miR-7-5p

As a tumor suppressor, miR-7-5p was markedly downregulated in radiation-resistant colorectal cancer cell lines (HCT116-R and RKO-R), and its diminished expression was significantly correlated with adverse prognosis in colorectal cancer patients.

miR-7-5p directly targets the 3′UTR of *KLF4* to diminish its expression, thereby attenuating the stemness properties of CRC cells and increasing their sensitivity to radiation therapy. KLF4 is a transcription factor linked to cancer cell stemness, facilitating self-renewal and drug resistance in cancer cells. Conversely, the overexpression of miR-7-5p attenuated this effect.

In addition, the administration of miR-7-5p mimics through nanoparticles markedly suppressed tumor proliferation and increased the radiosensitivity of CRC cells [34].

#### 2.2.2. miR-29b-3p

miR-29b-3p significantly influences the regulation of cancer cell stemness and tumor radioresistance. Expression of miR-29b-3p was observed to be downregulated in 3D cultured A549 (human lung cancer cells) and MCF7 (human breast cancer cells). The overexpression of miR-29b-3p markedly increased the radioresistance of 3D-cultured cells, diminished clonogenic capacity, and amplified radiation-induced DNA damage. Furthermore, the overexpression of miR-29b-3p diminished the percentage of CD133+ cells and attenuated the stemness attributes of the cells.

By targeting on the oncogenes *DNMT3B, PIK3R1, AKT2, and Bcl-2*, which are integral to DNA damage repair, cellular survival, and proliferation. miR-29b-3p impedes DNA damage repair and cellular viability, consequently augmenting radiosensitivity. Moreover, elevated levels of miR-29b-3p correlate with a positive prognosis in individuals with lung adenocarcinoma and breast cancer, suggesting its potential as a biomarker for forecasting response to radiation therapy [35].

#### 2.2.3. miR-339-5p

miR-339 has been discovered as a crucial factor in the radiosensitization of esophageal squamous carcinoma (ESCC). Cohort-based studies of ESCC have identified that miR-339-5p participates in stem cell division and DNA damage checkpoint signaling pathways. It suppresses stemness traits of ESCC cells by targeting the deubiquitinating enzyme (USP8), diminishes the expression of stemness-related markers such as SOX2 and NANOG, decreases the sphere-forming capacity of drug-resistant cells, and lowers the incidence of tumor formation in vivo. USP8 is a member of the ubiquitin-specific protease family. It is crucial in the initiation and progression of multiple malignancies, facilitating tumor cell proliferation, invasion, and metastasis [41,42].

Simultaneously, miR-339-5p can facilitate the DNA damage response and amplify radiation-induced DNA damage, thereby augmenting the radiosensitivity of ESCC cells. In light of the restricted miRNA recycling cycle and inadequate tumor targeting capability, the research additionally devised a multifunctional nanoplatform utilizing bismuth sulfide nanoflowers (Bi@PP) for the effective delivery of miR-339, thereby augmenting its prospective application in radiation therapy. In conclusion, these studies offer a significant theoretical foundation for the advancement of miRNA-based radiosensitization strategies [36].

#### 2.2.4. miR-449a

miR-449a, a tumor suppressor, markedly increases the sensitivity of prostate cancer cells to ionizing radiation by targeting c-Myc in prostate cancer. c-Myc is a nuclear phosphoprotein encoded by a proto-oncogene, and its dysregulation propels cancer progression by multifaceted reprogramming of cellular activity [43].

It was demonstrated that miR-449a expression was upregulated and c-Myc expression was downregulated following radiation exposure. miR-449a directly suppressed *c-Myc* expression by targeting its 3′-UTR, thereby modulating the Cdc25A/Cdc2/CyclinB1 cell cycle signaling pathway, resulting in G2/M phase cell arrest and augmenting radiotherapy-induced growth inhibition and cell cycle blockade [37].

#### 2.2.5. miR-495

In nasopharyngeal carcinoma cell lines, miR-495 exhibited an inverse correlation with GRP78 expression. In radioresistant samples, the expression of miR-495 was diminished, whereas the expression of GRP78 was elevated.

miR-495 was identified as a target for the 3′UTR of *GRP78*, consequently diminishing the expression of GRP78. GRP78 is a protein linked to tumor radioresistance, while miR-495 increases tumor radiosensitivity by inhibiting GRP78 activity.

Simultaneously, miR-495 was implicated in the regulation of epithelial-mesenchymal transition (EMT)-associated protein expression. The upregulation of miR-495 caused a downregulation of GRP78, vimentin, and N-cadherin, alongside an upregulation of E-cadherin, thereby diminishing the epithelial-mesenchymal transition (EMT) phenotype; conversely, the inhibition of miR-495 produced the opposite effect.

In summary, miR-495 significantly enhances the radiosensitivity of nasopharyngeal carcinoma by targeting GRP78 and modulating the expression of EMT-related proteins [38].

## 3. Regulation of the Self-Renewal Capacity of CSCs by miRNAs

Self-renewal of CSCs refers to the asymmetric division of CSCs in which a CSC divides into two distinct daughter cells: one retains the capacity for self-renewal while the other differentiates into a non-stem cell with a specific function (e.g., a tumor cell).This type of division helps to maintain the stability of the CSC population while generating the heterogeneous cells that make up the body of the tumor. This ability is a central feature that distinguishes CSCs from other tumor cells and underlies their key role in tumor recurrence and drug resistance [3,5].

Consequently, we can impede tumor progression by obstructing the self-renewal of CSCs. MicroRNAs are crucial in the self-renewal capacity of CSCs, modulating their maintenance, differentiation, and apoptosis through various mechanisms, thus influencing tumorigenesis and development. Table 2 and Figure 1B demonstrate the molecular mechanisms by which multiple miRNAs regulate the self-renewal of CSCs.

### 3.1. miR-2117

miR-2117, functioning as a tumor suppressor, inhibits the self-renewal of CSCs by directly repressing the transcription factor SOX2 in sorted EpCAM+ or CD24+ primary hepatocellular carcinoma (HCC) cells.

SOX2 is a constituent of the high mobility group of transcription factors crucial for sustaining stem cell pluripotency and self-renewal. The dysregulation of SOX2 has been linked to the progression and metastasis of various cancers, including HCC, where it plays a role in preserving CSC properties [58]. The downregulation of miR-2117 in LCSCs was demonstrated to elevate SOX2 expression, thereby enhancing the self-renewal ability and tumorigenesis of LCSCs. Conversely, the overexpression of miR-2117 reduced SOX2 levels, thereby impairing the self-renewal capacity of LCSCs [44].

Consequently, the overexpression of miR-2117 can suppress the self-renewal capacity of LCSCs and may serve as a potential therapeutic target for hepatocellular carcinoma treatment.

### 3.2. miR-194

miR-194 has been identified as a crucial regulator of self-renewal in various CSCs.

In HCC, the expression of miR-194 is diminished in chemotherapy-resistant HCC cells and in EpCAM- or CD133+ LCSCs. miR-194 suppresses the self-renewal and proliferation of LCSCs by directly targeting Ras-related C3 botulinum toxin substrate 1 (*RAC1*). Rac1 is a member of the Rac subfamily of Rho family GTPases and is integral to numerous cellular processes, which are diminished by the overexpression of miR-194.

In esophageal cancer (EC), miR-194 diminishes the self-renewal capacity of EC stem cells by targeting *PRC1*, thereby inhibiting the Wnt/β-linker signaling pathway. PRC1, a microtubule-binding and bundling protein, exhibits aberrant expression that promotes malignant growth in several malignancies [59]. PRC1 engages with the β-catenin disruption complex, which includes the APC protein, thereby undermining APC stability, facilitating β-catenin’s nuclear translocation, and stimulating Wnt target gene activation [60]. The activation of the Wnt/β-linker signaling pathway enhances self-renewal, proliferation, and invasion of EC stem cells, while the overexpression of miR-194 suppresses this pathway by silencing PRC1 [46].

### 3.3. miR-342-3p

In LCSCs, miR-342-3p suppresses self-renewal and tumorigenicity by targeting the HDAC7/PTEN axis.

HDAC7 and PTEN are crucial in regulating the self-renewal of LCSCs. *HDAC7*, a multifaceted transcriptional co-regulator that enhances cell proliferation and stem cell characteristics by modulating the tumor microenvironment, was markedly increased in LCSCs [61]. *PTEN* as a tumor suppressor [62] impeded the self-renewal capacity and non-anchorage-dependent growth potential of LCSCs while diminishing the expression of stemness-associated genes, including CD44, ALDH1, Bmi1, Sox2, and Oct4, thus attenuating LCSC stemness. The overexpression of HDAC7 suppressed PTEN expression.

The induction of miR-342-3p expression in LCSCs markedly suppressed HDAC7 expression, and the inhibition of HDAC7 resulted in enhanced histone H3 acetylation and upregulation of PTEN, subsequently inhibiting the self-renewal of LCSCs. The significant function of the miR-342-3p/HDAC7/PTEN axis in modulating LCSC characteristics was emphasized [47].

### 3.4. miR-130b-5p

In cervical cancer (CC) tissues, the expression of miR-130b-5p is diminished, while the expression of its target gene *ELK1* is elevated.

ELK1, an ETS transcription factor, functions as a transcriptional activator. The expression level correlates with the self-renewal capacity, proliferation, apoptosis, and other biological behaviors of cancer cells. Its aberrant expression frequently facilitates tumorigenesis.

The overexpression of miR-130b-5p functioned as a tumor suppressor in cervical cancer by targeting *ELK1*, diminishing the self-renewal capacity of cervical cancer stem cells and lowering the expression of stemness-associated proteins (OCT4, Sox2, and Nanog) [48].

### 3.5. miR-203

In glioblastoma, miR-203 expression was diminished in CD133+ cancer stem cells (CSCs) relative to non-CSCs, and its overexpression markedly decreased the self-renewal ability of glioblastoma stem cells [49].

In colorectal cancer, miR-203 suppresses the stemness of cancer cells by targeting *GATA6*, a crucial transcription factor for sustaining intestinal stem cells. Conversely, the overexpression of miR-203 diminished the characteristics of colon CSCs [50].

In leukemia, miR-203 expression is diminished in CD34 + AML cells, directly targeting *survivin* and *Bmi-1* to impede the self-renewal ability of leukemia stem cells. Survivin, a member of the inhibitor of the apoptosis (IAP) protein family, is significantly overexpressed in numerous human malignancies. Bmi-1, the multicombinomics proteome B-cell-specific Moloney murine leukemia virus integration site 1, is linked to the self-renewal of adult stem cells, and its silencing results in cell cycle arrest and senescence. The overexpression of miR-203 inhibits two critical factors associated with CSC self-renewal and drug resistance, consequently suppressing the proliferation and self-renewal of leukemia stem cells [51].

Collectively, these studies underscore the function of miR-203 as an inhibitor of CSCs, potentially impeding tumor development by suppressing CSC-driven tumor progression, recurrence, and consequently, tumorigenesis.

### 3.6. miR-630

miR-630 is markedly downregulated in CD44+/CD24+/ESA+ pancreatic cancer stem cells (PCSCs), while the expression of its target gene *PRKCI* is upregulated. PRKCI, or protein kinase C iota, is the encoded product of an atypical subclass within the protein kinase C (PKC) gene family. It is classified as an oncogene owing to its abnormal expression in cancer.

Research indicates that targeting *PRKCI* with miR-630 suppresses the activation of the Hedgehog signaling pathway, thereby impeding the self-renewal, proliferation, and tumorigenic potential of cancer stem cells while concurrently downregulating the expression of stem cell markers Oct4 and Nanog [52].

### 3.7. miR-486-5p

miR-486-5p inhibits the PI3K/Akt signaling pathway by targeting *p85*, thereby significantly reducing the proportion of CD133^+^ LCSCs and ultimately impairing their self-renewal capacity. P85 is a regulatory subunit of PI3K and an essential element of the PI3K/Akt signaling pathway. It activates the Akt signaling pathway through its interaction with the PI3K catalytic subunit, thereby enhancing cell survival and self-renewal. The overexpression of miR-486-5p counteracted the effects mediated by *P85*.

Moreover, the administration of miR-486-5p through lipid nanoparticles may further augment its therapeutic efficacy in vivo by markedly decreasing tumor volume and the fraction of CD133^+^ cells. Consequently, miR-486-5p is pivotal in the self-renewal of lung cancer stem cells via the p85/Akt signaling pathway, and the lipid nanoparticle delivery system offers a novel strategy for its clinical application, anticipated to serve as a potential target for lung cancer treatment [53].

In other cancer types, the mechanism of action of miR-486-5p varies. In glioblastoma (GBM), miR-486-5p augments the self-renewal capacity of GBM stem cells by targeting *PTEN* and *FOXO1*, thereby inhibiting the tumor suppressor network regulated by Sox2 [54]. In hepatocellular carcinoma, miR-486-5p diminishes Sirt1 expression by binding to its 3′UTR, thereby inhibiting the expression of stem cell-related genes (e.g., SOX2, OCT4, and CD13) and ultimately suppressing the self-renewal capacity of hepatocellular carcinoma cancer stem cells (CSCs) [55].

In summary, miR-486-5p modulates the self-renewal capacity of cancer stem cells by influencing various signaling pathways and molecular targets across different cancer types, thereby offering novel targets and strategies for cancer treatment.

### 3.8. miR-122-5p

In the CaSki cervical cancer cell line, the expression of miR-122-5p is diminished, whereas UCA1 and SOX2 are significantly upregulated. miR-122-5p, a small RNA with tumor suppressor properties, significantly contributes to the progression of cervical cancer.

miR-122-5p can suppress the self-renewal capacity of cervical cancer stem cells by targeting the 3′UTR of *SOX2*, thereby significantly diminishing their self-renewal potential. Long non-coding RNA UCA1 functions as a competitive endogenous RNA (ceRNA) by binding to miR-122-5p, thereby diminishing its degradation of SOX2 mRNA. This interaction results in elevated SOX2 expression levels and sustains the self-renewal capacity of CSCs [56].

The interplay between miR-122-5p and UCA1 affects the self-renewal and tumor advancement of cervical cancer stem cells through the modulation of SOX2 expression. This study elucidates the regulatory mechanism of the miR-122-5p/UCA1/SOX2 axis in the self-renewal of cervical cancer stem cells, offering a significant theoretical foundation for the advancement of novel therapeutic targets.

### 3.9. miR-182

Expression levels of miR-182 are diminished in acute myeloid leukemia (AML) and correlate with unfavorable prognosis. In a mouse model of MLL-AF9-induced acute myeloid leukemia (AML), diminished miR-182 expression expedites AML progression by enhancing the prevalence and self-renewal potential of leukemia stem cells (LSCs), thereby diminishing overall survival.

Research indicates that miR-182 impedes the self-renewal of LSCs by directly interacting with the 3′UTR sections of *BCL2* and *HOXA9*, thereby diminishing the protein production of these genes. BCL2 is an anti-apoptotic protein that prevents programmed cell death, is overexpressed in multiple malignancies, and is linked to tumorigenesis, progression, therapy resistance, and diminished overall survival [63]. HOXA9 is a crucial molecule in development and carcinogenesis, recognized as a significant oncogene in hematological malignancies, with its aberrant expression being a notable characteristic of several acute leukemias [64,65].

Elevated DNA methylation of the pri-miR-182 promoter inhibits miR-182 expression, whereas diminished miR-182 levels augment BCL2 and HOXA9 expression, facilitating LSC self-renewal and expediting AML progression. Significantly, miR-182 exerts negligible influence on the self-renewal ability of normal hematopoietic stem cells (HSPCs). Consequently, miR-182 presents potential as a therapeutic target for AML [57].

## 4. Regulation of Migration and Invasive Potential of CSCs by miRNAs

**Figure 2:**
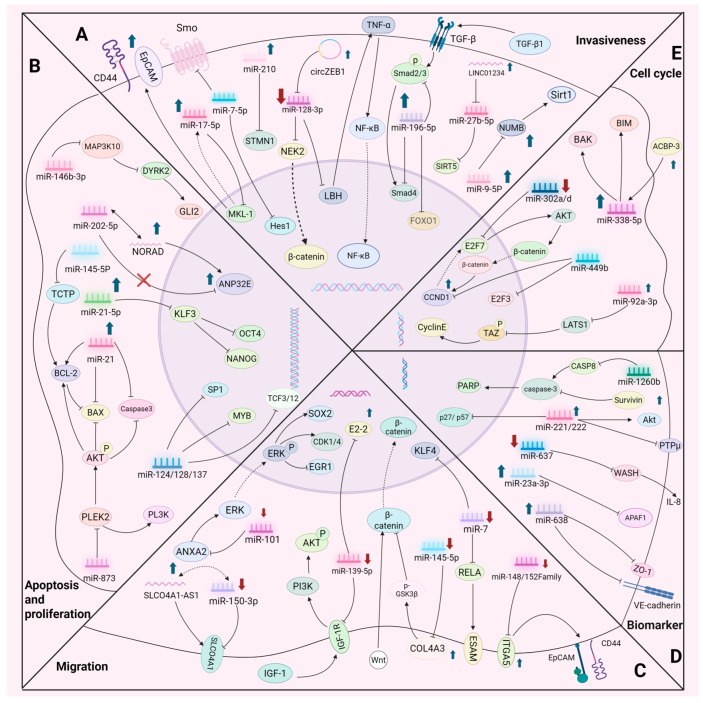
Molecular mechanisms by which miRNAs regulate invasiveness, proliferation, apoptosis, and metastasis; act as biomarkers; and influence the cell cycle in CSCs. (**A**) MiR-128-3p downregulation activates Wnt signaling pathway and boosts NEK2 expression. The FUS/circZEB1/miR-128-3p/LBH feedback loop contributes to CSC malignancy via TNF-α-mediated NF-κB signaling. Through miR-27b-5p adsorption, lINC01234 increases CSC invasiveness and SIRT5 expression. miR-7-5p reduces Smo and Hes1 inhibition and promotes Hedgehog signaling. CSC migration and invasiveness are adversely correlated with miR-9-5p and NUMB expression. miR-210 inhibits STMN1 expression to impair cellular flexibility and enhance CSC invasiveness. MKL-1 targets CD44, EpCAM, and miR-17-5p promoters to regulate CSC characteristics, while miR-17-5p inhibits MKL-1 production, creating a negative feedback loop. miR-196a-5p inhibits Smad4 and FOXO1 to increase CSC invasiveness. (**B**) Downregulating miR-145-5p increased TCTP and BCL-2 and decreased BAX and CASP3 expression, lowering CSC apoptosis. miR-146b-3p targeted MAP3K10 to regulate DYRK2 and GLI2 expression, which affected the Hedgehog signaling pathway. miR-21/miR-21-5p upregulated OCT4, SOX2, NANOG, BCL-2, and CCND1 while inhibiting BAX, KLF3, and CASP3, which promoted CSC proliferation and reduced apoptosis. miR-873 dysregulation increased PLEK2 expression, which stimulated the PI3K/AKT pathway and decreased CSC apoptosis. By competitively binding to miR-202-5p, lncRNA NORAD enhances the expression of its target gene ANP32E, which increases CSC proliferation and decreases apoptosis. (**C**) MiR-101 downregulation enhances ANXA2 expression, activates the ERK pathway, and suppresses EGR1 expression, which negatively feedbacks on miR-101 transcription. miR-7 deregulation increases KLF4 and RELA expression, which upregulates ESAM and boosts CSC migration. Downregulation of miR-148/152 family members enhances ITGA5 expression and CD44^+^EpCAM. Downregulation of miR-139-5p promotes E2-2 and IGF-1R expression, activating Wnt/β-catenin/TCF7L2 and IGF-1/IGF-1R pathways for CSC migration. Downregulation of miR-145-5p led to increased COL4A3 expression and Wnt/β-catenin pathway activation. SLCO4A1-AS1 competes with miR-150-3p to upregulate SLCO4A1 and enhance CSC migration. (**D**) Downregulation of miR-637 increases WASH, IL-8, Nanog, and SOX4/9 expression. miR-638 blocks ZO-1 and VE-cadherin. miR-1260b suppresses CASP8, caspase-3, and PARP and enhances cancer cell proliferation and metastasis. miR 23a-3p suppresses APAF1. miR-221/222 promotes Akt expression and suppresses p27, p57, and PTPµ expression. (**E**) miR-302a/d downregulation increases E2F7 and its downstream signaling pathway, speeding CSCs from G1 to S phase. miR-449b promotes G1-to-S phase cell transition by downregulating CCND1 and E2F3 expressions. miR-92a-3p inhibits LATS1 to upregulate TAZ and downregulate E-cadherin and facilitates G1-to-S-phase cell transition. miR-338-5p promotes G0/G1-to-S and G2/M cell transition by upregulating BAK and BIM.

The invasiveness and migratory characteristics of CSCs are essential attributes they develop during tumor progression. By activating the epithelial–mesenchymal transition (EMT) process and associated signaling pathways (e.g., Notch, Hedgehog, Wnt, etc.), CSCs can breach the basement membrane, infiltrate adjacent tissues, and disseminate to distant sites to establish metastatic foci via the bloodstream or lymphatic system [66,67,68,69,70]. These characteristics render cancer stem cells pivotal in tumor recurrence and therapeutic resistance. miRNAs may be modulating the invasiveness and migratory ability of CSCs through multiple mechanisms. Table 3 and Figure 2A,C demonstrate the molecular mechanisms by which multiple miRNAs regulate the migratory ability and invasiveness of CSCs.

### 4.1. Regulation of the Migratory Capacity for CSCs by miRNAs

#### 4.1.1. miR-101

The expression of miR-101 is diminished in CD133 + LCSCs, which suppresses malignant characteristics, including LCSC migration, by targeting *ANXA2*.

ANXA2, also known as Annexin A2, is a protein that plays a role in cell cycle progression and cellular migration. Extracellular signal-regulated kinase (ERK) is a signaling molecule whose activation amplifies the characteristics of CSCs by upregulating SOX2 and cell cycle-associated kinases, thereby further augmenting CSC properties. Phosphorylation of ERK concurrently suppresses the expression of EGR1, a transcriptional activator of miR-101, resulting in the inhibition of miR-101 transcription due to EGR1’s suppression by ERK.

In LCSCs, the activation of miR-101 expression suppresses the mRNA and protein levels of ANXA2, consequently inhibiting the activation of the ERK pathway and diminishing the expression of associated molecules such as SOX2, thereby impeding the migration of LCSCs. The novel regulatory circuit miR-101/ANXA2/EGR1 presents a new therapeutic target for liver cancer treatment [71].

#### 4.1.2. miR-7

miR-7 functions as a tumor suppressor in breast cancer stem cells (BCSCs), diminishing their metastatic capability.

Research indicates that miR-7 is markedly downregulated in metastatic breast cancer stem cells, especially in those with increased potential for brain and bone metastases. The downregulation correlates with elevated expression levels of the transcription factor KLF4. Increased KLF4 expression augments the self-renewal ability and invasiveness of cancer stem cells (CSCs) [88], but miR-7 overexpression markedly diminishes CSC metastatic activity by directly interacting with the 3′UTR of *KLF4* mRNA. In vivo experiments revealed that miR-7 overexpression markedly diminished brain metastasis while exerting little impact on bone metastasis of BCSCs, suggesting its particular regulatory role on brain metastasis through KLF4 modulation [72]. Furthermore, miR-7 diminishes the metastatic capacity of cancer stem cells (CSCs) via targeting RELA to suppress the production of endothelial-cell-selective adhesion molecule (ESAM) [73].

#### 4.1.3. miR-148/152 Family

The expression of miR-148/152 family members is diminished in gastric cancer stem cells (GCSCs), and their expression induction impedes cell migration in GCSCs.

ITGA5, or integrin α5, is a constituent of the integrin adhesion molecule family, which is significantly linked to the initiation, advancement, and metastasis of tumors. ITGA5 is significantly expressed in gastric cancer stem cells, and its elevated expression correlates with reduced survival rates in gastric cancer patients. The overexpression of miR-148/152 family members may suppress the activation of the Wnt signaling pathway by directly targeting *ITGA5*, thereby diminishing the nuclear accumulation of β-catenin in GCSCs, reducing the expression of cell surface markers CD44 and EpCAM, and ultimately inhibiting cell migration. In conclusion, members of the miR-148/152 family may serve as potential targets for the treatment of gastric cancer metastasis [74].

#### 4.1.4. miR-139-5p

miR-139-5p demonstrated substantial regulatory effects in suppressing the migration of CSCs.

Research demonstrated that miR-139-5p markedly diminished the migratory capacity of colon cancer stem cell-like cells by targeting and suppressing the expression of E2-2, a pivotal transcription factor within the Wnt/β-catenin signaling pathway, whose overexpression is intricately linked to EMT and tumor metastasis in various cancers. In colon cancer stem cell-like cells, miR-139-5p directly targets the 3′-UTR of E2-2, thereby inhibiting its protein expression. This modulation subsequently influences the transcription of downstream effectors in the Wnt signaling pathway, ultimately compromising the migratory capacity of colon cancer stem cell-like cells [75].

Moreover, miR-139-5p suppressed the migration of hemangioma stem cells (HemSCs) by targeting the insulin-like growth factor 1 receptor (*IGF-1R*), which plays a role in the regulation of the IGF-1/IGF-1R signaling pathway [76]. The findings indicate that miR-139-5p is crucial in regulating the migration of various cancer stem cells and may represent a viable target for tumor therapy.

#### 4.1.5. miR-145-5p

In LCSCs, the downregulation of miR-145-5p expression is significantly associated with the malignant traits of tumors, and its mechanism for regulating the migratory capacity of LCSCs primarily involves targeting Collagen Type IV Alpha 3 Chain (*COL4A3*).

COL4A3 is a protein prominently expressed in LCSCs and plays a role in cell migration. COL4A3 overexpression suppresses GSK-3β activity by facilitating the phosphorylation of the Ser9 site of GSK-3β(p-GSK-3βS9), which encourages the nuclear translocation of β-catenin and activates the Wnt/β-catenin signaling pathway, thereby augmenting the migratory capacity of LCSCs.

Moreover, COL4A3 diminished cellular autophagy by modulating the GSK3β/Gli3/VMP1 axis, thereby enhancing the migration of LCSCs. Therefore, the analysis of COL4A3 provides a new molecular mechanism and potential target for hepatocellular carcinoma therapy [77].

#### 4.1.6. miR-150-3p

The expression of miR-150-3p is diminished in colorectal cancer stem cells (CCSCs). Research indicates that the overexpression of miR-150-3p can suppress its own expression by directly targeting the 3′-UTR of *SLCO4A1*, therefore modulating the migratory ability of CCSCs.

SLCO4A1 is a transport protein implicated in cell migration, and its elevated expression is strongly correlated with the advancement of several malignancies. In colon cancer cells, SLCO4A1 is markedly upregulated, along with elevated expression of its antisense RNA (SLCO4A1-as1). SLCO4A1-as1 can competitively bind to miR-150-3p, mitigating its inhibitory impact on SLCO4A1, resulting in enhanced SLCO4A1 expression and facilitating CCSC migration [78].

In conclusion, miR-150-3p suppresses the migratory ability of CCSCs by targeting SLCO4A1, with this regulatory impact being competitively influenced by SLCO4A1-as1. This discovery offers novel insights into the molecular pathways underlying colon cancer.

### 4.2. Regulation of Cancer Stem Cell Invasiveness by miRNAs

#### 4.2.1. miR-128-3p

As a tumor suppressor, miR-128-3p modulates downstream pathway activation by targeting *NEK2/LBH* and impedes the invasiveness of associated cancer stem cells.

In breast cancer, miR-128-3p expression is diminished in BCSCs, and its overexpression in these cells can inhibit the Wnt signaling pathway both in vivo and ex vivo by directly targeting the 3′-UTR of NIMA-associated kinase 2(*NEK2*). This results in reduced NEK2 expression, subsequently causing inactivation of the Wnt/β-linker pathway and significantly diminishing the invasiveness of BCSCs [79].

In glioblastoma, circZEB1 functions as a sponge for miR-128-3p, sequestering it and diminishing its inhibitory impact on the expression of the transcriptional cofactor LBH. This, in turn, enhances the transcription of tumor necrosis factor-α(TNF-α), subsequently activating the NF-κB signaling pathway and facilitating glioma progression. Inhibition of LBH expression by miR-128-3p resulted in decreased TNF-αexpression and secretion, alongside suppression of the NF-κB signaling pathway, thereby reducing the invasiveness of GSCs. circZEB1 functions as a sponge adsorbent, potentially facilitating glioblastoma progression [80].

These findings underscore the tumor suppressor function of miR-128-3p in mitigating the invasiveness of cancer stem cells via various signaling pathways, indicating its potential as a therapeutic target for cancers characterized by elevated CSCs activity.

#### 4.2.2. miR-9-5p

miR-9-5p is an oncogenic microRNA linked to unfavorable prognosis in various malignant tumors, and it markedly enhances the malignant characteristics of CSCs in prostate cancer (PCa).

Levels of miR-9-5p are elevated in CD44^+^ prostate cancer stem cells (PCSCs) and exhibit a negative correlation with the expression of NUMB, a tumor suppressor in PCSCs. NUMB is an evolutionarily conserved protein that is broadly expressed in mammalian tissues, and recent findings suggest that NUMB may exert tumor suppressor effects across various tumor types [81].

The direct targeting of miR-9-5p to the 3′-UTR of *NUMB* mRNA led to its downregulation and increased cell invasion of PCSCs.

#### 4.2.3. miR-210

miR-210 diminishes the cellular rigidity of colorectal cancer stem cells (CRCSCs) by targeting Stathmin1 (*STMN1*), thereby enhancing their invasiveness, a process that is independent of epithelial–mesenchymal transition (EMT).

miR-210 was observed to be upregulated in CRCSCs, resulting in diminished cell elasticity and increased cell deformation through the inhibition of STMN1 expression, thereby softening the cells and augmenting the migration and invasiveness of CRCSCs.

The miR-210 high expression and STMN1 low expression pattern (miR-210High/STMN1Low) correlated with liver metastasis and unfavorable prognosis in colorectal cancer patients, suggesting its potential as a prognostic marker [82].

#### 4.2.4. miR-27b-5p

miR-27b-5p, functioning as a tumor suppressor, is expressed at low levels in ovarian cancer tissues and correlates with unfavorable prognosis and lymphatic metastasis.

In ovarian cancer, miR-27b-5p suppresses the invasive capacity of ovarian cancer stem cells (OCSCs) by directly targeting *SIRT5*. SIRT5 is elevated during cellular transition, facilitating cell proliferation and cancer. SIRT5 is significantly expressed in human breast cancers and is linked to unfavorable patient prognosis [88]. LINC01234, a miR-27b-5p sponge, exhibited a negative correlation with the expression of miR-27b-5p in ovarian cancer tissues. LINC01234 effectively downregulated the activity of miR-27b-5p, subsequently promoting the expression of SIRT5 and markedly augmenting the invasive capacity of OCSCs [83].

In summary, miR-27b-5p significantly influences the invasiveness of OCSCs, and the identification of LINC01234 offers a novel target for precision therapy in ovarian cancer treatment.

#### 4.2.5. miR-17-5p

The expression level of miR-17-5p is positively associated with the invasiveness of gastric cancer stem cells (GCSCs) and augments their invasive potential. The overexpression of miR-17-5p markedly elevates the expression of stem cell markers CD44 and EpCAM in MGC-803 gastric cancer cells, augments tumor spheroid forming capacity, and promotes cellular resistance to chemotherapeutic agents. Significantly, the overexpression of MKL-1 yields analogous pro-stem effects.

Mechanistic investigations demonstrate that MKL-1 overexpression markedly elevates miR-17-5p expression levels. Furthermore, miR-17-5p can target and bind to the 3′ UTR region of *MKL-1* mRNA, thereby suppressing MKL-1 protein expression in in vitro tests. Conversely, in vivo investigations produced contrasting outcomes, revealing a synergistic elevation in the expression levels of miR-17-5p and MKL-1. The disparity between in vivo and in vitro findings indicates the existence of intricate regulation mechanisms, possibly involving circRNA or lncRNA through competitive endogenous RNA (ceRNA) mechanisms that attenuate the activity of miR-17-5p [84].

In conclusion, miR-17-5p and MKL-1 establish a bidirectional regulatory network in vivo, together augmenting the invasive properties of GCSCs.

Indeed, miRNAs play a crucial role in other CSCs by modulating their invasiveness and migratory capabilities. For instance, the expression of miR-7-5p is diminished in CD44+ gastric cancer cell lines, which impedes the function of Smo (a pivotal protein in the Hedgehog signaling pathway) and Hes1 (a downstream target gene of the Notch signaling pathway) by targeting these two signaling pathways, consequently decreasing the colony formation and invasive capacity of GCSCs [85]. Moreover, miR-196a-5p exhibited elevated expression in GCSCs and suppressed its expression by targeting *Smad4*, which facilitated TGF-β-induced epithelial-mesenchymal transition (EMT), consequently augmenting the invasive and migratory capabilities of GCSCs [86]. In GSCs, miR-196a-5p suppressed the expression of FOXO1 and its downstream gene MIIP by targeting the 3′-UTR of *FOXO1*, consequently augmenting the migratory and invasive capabilities of GSCs [87].

## 5. The Regulatory Function of miRNAs in the Proliferation and Apoptosis of CSCs

Concurrently, CSCs maintain tumor heterogeneity and promote growth through the upregulation of anti-apoptotic proteins, inhibition of death receptors (Fas/TRAIL-R), activation of the NRF2-KEAP1 antioxidant pathway, and autophagy, thereby evading apoptotic signaling and resisting DNA damage induced by radiotherapy, ultimately resulting in tumor resistance and recurrence [89,90]. In recent years, miRNAs, as pivotal regulatory molecules of gene expression, have exhibited multifaceted regulatory functions in the maintenance of stemness, proliferation, and apoptosis dysregulation of CSCs, emerging as a promising research avenue to overcome the existing limitations of tumor therapy. Table 4 and Figure 2B demonstrate the molecular mechanisms by which multiple miRNAs regulate the proliferation and apoptosis of CSCs.

### 5.1. miR-145-5p

miR-145-5p functions as a tumor suppressor in GSCs. miR-145-5p was observed to be expressed at a diminished level in primary GSCs; however, its upregulation markedly suppressed the proliferative capacity of GSCs and enhanced the apoptosis rate. In multiple human glioma cell lines, miR-145-5p suppressed stem cell proliferation by directly targeting the translation-regulated tumor protein (TCTP).

TCTP exhibits elevated expression in multiple cancer types and correlates with unfavorable prognosis. It demonstrates anti-apoptotic properties in neoplastic cells, obstructing apoptosis and facilitating tumor proliferation [107,108]. The elevated expression of TCTP is essential for preserving the identity of GSCs and for tumorigenesis. Consequently, the miR-145-5p/TCTP axis holds significant research value in miRNA-targeted glioma therapy [91].

### 5.2. miR-146b-3p

miR-146b-3p demonstrates tumor-suppressive properties in pancreatic cancer stem cell-like cells (P-CSCs). Research demonstrated that miR-146b-3p suppressed cell proliferation and induced apoptosis by targeting the 3′-UTR of MAP3K10 mRNA in pancreatic cancer cell lines Mia-paca-2 (CSChigh) and BxPC-3 (CSClow).

MAP3K10 is a plasma membrane protein kinase that regulates the activation of the Hedgehog signaling pathway. MAP3K10 interacts with DYRK2, which subsequently enhances the expression of GLI2 and facilitates the proliferation of P-CSCs.

The overexpression of MAP3K10 in P-CSCs enhanced cell proliferation by downregulating miR-146b-3p. In summary, miR-146b-3p disrupted the progression of P-CSCs and impeded cellular proliferation by targeting *MAP3K10* [92].

### 5.3. Conjunction of miR-124, miR-128, and miR-137

Research demonstrated that the combination of miRNAs (miR-124, miR-128, and miR-137) is an effective approach for suppressing the proliferation and survival of glioma stem cells (GSCs).

These miRNAs impede the stem cell characteristics of cells by targeting various transcription factors (e.g., SP1, MYB, TCF3, and TCF12) and their regulatory networks, consequently diminishing the cell’s proliferative potential [93].

In conclusion, this miRNA combination therapy offers a novel and more efficacious therapeutic approach for targeting GSCs.

### 5.4. miR-21

miR-21 influences apoptosis in oral cancer stem cells (OCSCs) through the regulation of various essential genes and signaling pathways.

Research indicates that miR-21 is markedly upregulated in CD44^+^ OCSCs, where it suppresses apoptosis by targeting and downregulating the expression of *BAX* and *Caspase 3* genes, concurrently enhancing the expression of the anti-apoptotic gene *Bcl-2*.

Bcl-2 homologous antagonist X (BAX) is a pro-apoptotic protein within the Bcl-2 protein family. It induces an elevation in mitochondrial membrane permeability via pore formation, liberates cytochrome c, and triggers a subsequent caspase cascade, ultimately resulting in apoptosis. Cysteine aspartate protease 3 (Caspase 3) is a pivotal executive caspase enzyme situated downstream in the apoptotic cascade. The overexpression of miR-21 promotes the survival of OCSCs by suppressing two pro-apoptotic genes and upregulating Bcl-2 to prevent apoptosis in OCSCs [94].

### 5.5. miR-873

Research indicates that miR-873 suppresses the PI3K/AKT signaling pathway by targeting Plekstrin-2 (*PLEK2*) in pancreatic cancer stem cells (PANCSCs), thereby markedly enhancing CSC apoptosis.

PLEK2 expression levels are consistently increased across multiple cancer types and are strongly correlated with tumor invasiveness, metastasis, and worse prognosis. It exerts its carcinogenic effects via several signaling pathways in numerous malignancies [109].

miR-873 diminished PLEK2 expression by targeting its 3′UTR, subsequently inhibiting the activation of the PI3K/AKT signaling pathway. The suppression of this pathway results in elevated levels of pro-apoptotic proteins cleaved caspase-3 and Bax, alongside a reduction in the anti-apoptotic protein Bcl-2, thereby facilitating the apoptosis of CSCs. miR-873 may serve as a novel molecular target for pancreatic cancer therapy [95].

### 5.6. miR-21-5p

miR-21-5p modulates apoptosis in pancreatic cancer stem cells (PCSCs) by targeting Kruppel-like factor 3 (*KLF3*), a transcription factor implicated in an oncostatic function across various malignancies, including the regulation of apoptosis.

The upregulation of miR-21-5p in extracellular vesicles (EVs) derived from M2 macrophages was found to suppress KLF3 expression, thereby augmenting the anti-apoptotic capacity of PCSCs and decreasing their sensitivity to apoptosis-inducing agents like perifosine. Conversely, the downregulation of miR-21-5p reinstated KLF3 expression and elevated the apoptosis rate of PCSCs.

In conclusion, miR-21-5p is essential for the survival of PCSCs, presenting a novel target for therapeutic intervention in pancreatic cancer [96].

### 5.7. miR-202-5p

miR-202-5p functions as a tumor suppressor and demonstrates reduced expression in pancreatic cancer tissues and cells, but the expression levels of NORAD and *ANP32E* are elevated.

ANP32E, a member of the acid nucleoprotein 32 family, participates in cell proliferation, carcinogenesis, and metastasis [110]. The overexpression of miR-202-5p suppresses the proliferation of pancreatic cancer stem cells (PCSCs) by targeting ANP32E and promotes their apoptosis. The long non-coding RNA NORAD can mitigate this inhibitory effect. NORAD functions as a “molecular sponge” for miR-202-5p in the cytoplasm, competing for binding and thereby inhibiting its regulatory effect on the target gene ANP32E. This results in elevated expression of ANP32E, thus augmenting the proliferative capability of PCSCs and suppressing their apoptosis [97].

## 6. Regulation of the Cell Cycle in Cancer Stem Cells by miRNAs

The cell cycle dynamics of CSCs are essential for their involvement in tumor initiation, treatment resistance, and recurrence. The cell cycle of CSCs is distinguished by unique dynamics, exhibiting a propensity to enter a quiescent (G0/G1) or slow-cycling state (quiescence) to evade destruction by conventional chemotherapy and radiotherapy targeting rapidly dividing cells. Quiescent (G0/G1) cells facilitate tumor dormancy and therapeutic resistance, whereas cycling (S/G2/M) cells enhance metastasis. This “dormant-like” characteristic enables them to endure prolonged durations and re-enter the proliferative cycle when prompted by the tumor microenvironment (e.g., hypoxia or inflammatory signals), facilitating tumor regeneration and metastasis [111,112,113,114].

miRNAs bidirectionally modulate the quiescence and proliferation of CSCs by targeting cell cycle regulatory proteins. The miRNA-mediated dynamic equilibrium is a crucial mechanism for drug resistance and tumor recurrence in CSCs, offering a potential therapeutic strategy for targeting cell cycle regulation in CSCs. Table 4 and Figure 2E demonstrate the molecular mechanisms by which multiple miRNAs regulate the cell cycle in CSCs.

### 6.1. miR-338-5p

miR-338-5p regulates the cell cycle of GCSCs, and its overexpression markedly influences cell cycle progression.

The overexpression of miR-338-5p diminished the population of GCSCs in the G0/G1 phase while augmenting their numbers in the S and G2/M phases, thereby inducing alterations in the cell cycle and suppressing the associated characteristics of GCSCs.

The alterations in the cell cycle offer a novel insight into the role of miR-338-5p in GCSCs and potential targets for formulating therapeutic approaches against GCSCs [98].

### 6.2. miR-302a/d

Research indicates that the inhibition of miR-302a/d expression obstructs cell cycle progression. The suppression of miR-302a/d caused a notable G1-phase arrest or postponed cell progression into the S phase in the hepatocellular carcinoma cell lines HepG2 and Huh7.

miR-302a/d suppresses cell cycle initiation in LCSCs by targeting E2F7. The overexpression of miR-302a/d markedly suppressed the proliferation of LCSCs and impeded the transition of the cell cycle from G1 to S phase by inhibiting E2F7 and its downstream AKT/β-catenin/CCND1 signaling pathway.

The overexpression of E2F7 activated the AKT1-cyclin D1 signaling pathway, thereby facilitating cell cycle progression. Conversely, the expression of miR-302a/d suppressed this effect [99].

### 6.3. miR-449b

Overexpression of miR-449b obstructed the transition of the cell cycle from G1 to S phase, while inhibition of miR-449b diminished the proportion of cells in the G1 phase.

Mechanistic investigations have demonstrated that miR-449b modulates the cell cycle progression of colorectal cancer stem cells (CRC-CSCs) via targeting the 3′ UTR regions of *CCND1* and *E2F3*. CCND1 is a pivotal component of the cyclin D family and functions as a principal regulator of the G1/S checkpoint [115]; E2F3 is a crucial activator of the S phase in proliferating cells, possessing DNA-binding ability that demonstrates cell cycle specificity [116]. Considering miR-449b’s suppressive effects on these two essential cell cycle regulatory elements, it may serve as a viable therapeutic target for colon cancer treatment [100].

### 6.4. miR-92a-3p

The expression of miR-92a-3p is elevated in cervical cancer tissues and facilitates cell cycle progression in cervical cancer stem cells (CCSCs) by targeting *LATS1*.

LATS1 is an essential element of the Hippo signaling pathway. The overexpression of LATS1 suppresses the proliferation of cervical cancer stem cells, leading to an increased percentage of cells in the G1 phase of the cell cycle and a reduced percentage of cells in the S phase. LATS1 suppresses the expression of cyclin E while enhancing the expression of p27. Cyclin E is essential for the G1/S phase transition, and the association of p27 with the cyclin E binary complex impedes its kinase activity, thus negatively regulating the cell cycle transition.

The overexpression of miR-92a-3p downregulates LATS1, resulting in the upregulation of cyclin E expression, which facilitates the transition of cells from the G1 phase to the S phase, thereby accelerating cell cycle progression and fostering malignant behavior in CCSCs [101].

## 7. miRNAs as Biomarkers in Cancer

MicroRNAs are a category of small, non-coding RNAs that significantly influence the onset, progression, and treatment of cancer. Research indicates that microRNAs display abnormal expression across various cancers, with their expression patterns closely associated with tumorigenesis, progression, prognosis, and therapeutic response. Recently, microRNAs have garnered significant attention for their considerable potential as biomarkers in cancer diagnosis and prognosis.

Exosomal microRNAs have emerged as a significant research focus for non-invasive cancer detection, owing to their stability in blood, urine, and other bodily fluids, thereby offering a novel approach for early screening and treatment monitoring of cancer [117,118].

It is anticipated that advancements in science and technology, along with comprehensive clinical trials, will enable microRNAs to significantly contribute to the early diagnosis, personalized treatment, and prognostic evaluation of cancer. Table 4 and Figure 2D demonstrate the role of various miRNAs as biomarkers in cancer cells.

### 7.1. miR-637

miR-637 demonstrates significant prognostic potential in ESCC. The expression level of miR-637 in tumor tissues was significantly reduced compared to normal tissues, and its diminished expression was strongly correlated with adverse pathological characteristics, including advanced TNM stage, poor tumor differentiation, and lymph node metastasis. Furthermore, diminished expression of miR-637 correlated with reduced overall survival in ESCC patients.

The expression level of miR-637 in the blood was significantly reduced and positively correlated with advanced TNM stage and lymph node metastasis. ROC curve analysis indicated that plasma miR-637 exhibited high diagnostic sensitivity and specificity (AUC = 0.911), implying its potential as a non-invasive biomarker for the diagnosis and prognostic evaluation of ESCC. Mechanistically, miR-637 impedes cancer stem cell properties by targeting the WASH/IL-8 pathway, thereby exerting its tumor suppressor effect. Consequently, miR-637 may function as both a potential prognostic biomarker for ESCC and a therapeutic target [102].

### 7.2. miR-638

MiR-638 has been identified as a potential prognostic marker for hepatocellular carcinoma. It originates from exosomes released by highly invasive hepatocellular carcinoma cells and facilitates intrahepatic metastasis by targeting VE-cadherin and tight junction protein 1 (ZO-1) in endothelial cells, thereby disrupting adhesion junctions between endothelial cells and enhancing vascular permeability.

The research indicated that elevated levels of exosome miR-638 in serum were markedly correlated with a heightened incidence of postoperative recurrence in patients, exhibiting a 2-year disease-free survival (DFS) rate of merely 47.1%, substantially lower than the 77.4% observed in the low-expression cohort. In both univariate and multivariate analyses, elevated expression of miR-638 was recognized as an independent prognostic indicator for disease-free survival in hepatocellular carcinoma patients [103].

Notwithstanding the limited sample size of this study, the promise of miR-638 as a prognostic biomarker offers a novel avenue for postoperative monitoring and risk evaluation of HCC patients.

### 7.3. miR-491-5p

miR-491-5p was found to be a potential diagnostic and prognostic marker for HNSCC. The study revealed that miR-491-5p was markedly upregulated in the plasma of patients with HNSCC, based on the analysis of extracellular vesicles (EVs) miRNA expression. The diagnostic model demonstrated a specificity of 100%, albeit with a low sensitivity of 46.6%.

More importantly, the dynamic change of miR-491-5p before and after treatment (ΔmiR-491-5p) was significantly correlated with the overall survival (OS) and disease-free survival (DFS) of patients and was an independent prognostic factor for OS and DFS in HNSCC patients.

This suggests thatΔmiR-491-5p could serve as a potential marker for monitoring treatment response and prognosis in HNSCC patients and help identify patients at high risk of relapse. Notwithstanding the restricted sample size and brief follow-up duration, the identification of miR-491-5p offers a novel avenue for liquid biopsy in HNSCC [119].

### 7.4. miR-1260b

Studies have shown that miR-1260b is a potential diagnostic and prognostic marker for the detection and prognostic evaluation of breast cancer. miR-1260b expression levels in breast cancer tissues were significantly correlated with clinicopathological features such as tumor size, TNM stage, and lymph node metastasis, and high expression of miR-1260b was correlated with worse overall survival (OS).

In plasma, the expression level of miR-1260b was significantly higher in breast cancer patients than in healthy controls, with an AUC value of 0.90, showing a high diagnostic value. Consequently, miR-1260b serves as both an independent prognostic factor and a potential diagnostic marker [104].

### 7.5. miR-221/222

As oncogenic factors, miR-221/222 were found to be independent prognostic markers in glioblastoma (GBM) patients. They demonstrated considerable prognostic significance in two independent cohorts (TCGA cohort and Heidelberg University Hospital cohort), with elevated expression markedly correlated with reduced overall survival (OS).

Specifically, high expression of miR-221 and miR-222 were significantly associated with shorter survival, and their high expression was positively correlated with poor prognosis and recognized as independent prognostic markers in multivariate analysis. Furthermore, elevated levels of miR-221/222 were correlated with enhanced invasiveness of tumor cells, potentially contributing to radiotherapy resistance in GBM cells.

In conclusion, miR-221/222 possess significant prognostic value in GBM and may serve as prospective therapeutic targets in the future [105].

### 7.6. miR-23a-3p

miR-23a-3p serves as a prospective biomarker for predicting platinum resistance and unfavorable prognosis in patients with high-grade plasmacytoid ovarian cancer (HGSOC). miR-23a-3p was significantly overexpressed in platinum-resistant patients and was associated with shorter progression-free survival (PFS).

Similarly, miR-23a-3p was significantly upregulated in ovarian cancer stem cell-like cells (OVA-BS4 spheroids), which are speculated to be possibly involved in tumor drug resistance mechanisms. Inhibition of miR-23a-3p in ovarian cancer increased APAF1 protein levels and enhanced the sensitivity of tumor cells to platinum drugs, whereas overexpression of miR-23a-3p led to decreased APAF1 expression and promoted the platinum resistance phenotype [106].

miR-23a-3p is not only an independent prognostic marker but may also affect apoptosis and platinum resistance of HGSOC cells by targeting APAF1, providing a new target for precision treatment of HGSOC.

## 8. The Cross-Cancer Perspective Includes the Commonalities and Specificities of Pivotal miRNAs in the Control of Cancer Stem Cell Functions

Upon integrating the prior comprehensive research of unique microRNAs that govern self-renewal, metastasis, apoptosis, and invasion in several cancer stem cell types (CSCs), we noted that numerous individual miRNAs behave as coregulators across multiple cancer contexts. This indicates the potentially conserved nature of their functions while emphasizing the complexity of heterogeneity among cancer species. Numerous prominent miRNAs are crucial in regulating CSC function across various malignancies. Table 5 shows the regulatory roles of various key miRNAs in multiple cancer CSCs.

For instance, members of the miR-34 family (e.g., miR-34a) have been extensively documented to demonstrate significant tumor suppressor effects in cancer stem cells across numerous malignancies, including breast [120,121], prostate [122,123], and colorectal [124] cancers. They impede self-renewal and metastasis, induce apoptosis, and augment chemosensitivity by directly targeting essential stem cell factors and anti-apoptotic proteins (e.g., NOTCH1, BCL2, SIRT1). Another pivotal regulator is miR-137, which frequently functions as a suppressor of stemness, inhibiting stemness characteristics such as self-renewal and metastasis of cancer stem cells (CSCs) by targeting genes including *DCLK1*, *SOX2*, *NANOG*, and *FUNDC1*, as well as the Wnt and SHH signaling pathways across various CSCs, including those in colon [125], pancreatic [126], glioblastoma [127] and breast [128] cancers. The functional conservation across several cancer types strongly indicates that the miR-34 family and miR-137 are crucial in suppressing CSC features, rendering them appealing broad-spectrum therapeutic targets.

It is significant that both oncogenic miRNAs exhibit cross-cancer effects, and the prominent “oncogenic miRNA (oncomiR)” miR-21 modulates cancer stem cell function across many malignancies. In oral [94], pancreatic [129], breast [130], and colon [131] cancers, miR-21 enhances cancer stem cell survival, proliferation, and invasion by targeting stemness markers, including OCT4, SOX2, CD133, and CD44, as well as signaling pathways such as Wnt and AKT/ERK1/2. Specifically, in colorectal malignancies, miR-21 can modulate cancer stem cell functionality across various cancer types. In colon cancer, miR-21 negatively regulates miR-145 and is pivotal in modulating the proliferation and differentiation of cancer stem cells, as well as the advancement of chemoresistance [131]. This indicates that various miRNAs often create synergistic or antagonistic combinatorial networks to meticulously regulate the nodes of essential signaling pathways.

The preceding section clearly indicates that several miRNAs exhibit both commonality and specificity in the regulation of CSC function. The similarity resides in the mechanism by which these miRNAs typically target particular anti-apoptotic proteins, stemness indicators, or signaling pathways, whilst the specificity is evident in the varying selection of targets across different cancer types or contexts. These specific targets frequently exhibit considerable overlap within the action networks of several miRNAs. The significant target overlap is essential for comprehending the intricacy and synergistic characteristics of miRNA-regulated CSC functional networks. The Wnt/β-catenin pathway is essential for preserving stem cell characteristics and is inhibited by several tumor suppressor miRNAs, including miR-137 [126], the miR-378 family [21], and miR-139-5p [75]. They inhibit pathway activity by directly targeting β-catenin or its essential cofactors (e.g., LEF1), receptors (e.g., FZD receptor), or regulatory proteins (e.g., APC, AXIN2). The BCL2 family of crucial anti-apoptotic proteins, besides being typical targets of miR-21 [94], is also influenced by miRNAs such as miR-29b-3p [35] and miR-182 [57], which together enhance the apoptosis sensitivity of cancer stem cells (CSCs).

Consequently, a comprehensive analysis of the aforementioned miRNA regulatory networks necessitates not only the identification of specific miRNA targets but also an understanding of the critical combinations of miRNAs that serve as regulators within a particular CSC context, their synergistic or antagonistic interactions with core node networks, and the dynamic alterations of these networks in response to therapeutic pressures, such as chemotherapy. This network approach is essential for devising more effective therapeutic techniques to target cancer stem cells (CSCs). The investigation and utilization of miRNAs as “network regulators” instead than just as single gene regulators is pivotal for the future advancement of comprehensive miRNA-based anti-CSC therapy.

## 9. Therapeutic Approaches Targeting miRNA Modulation of Cancer Stem Cell Activity

Targeting and modulating the expression of critical miRNAs in cancer stem cells has emerged as a promising anti-tumor treatment approach. This is mostly accomplished through two complimentary mechanisms: employing antagonists to suppress oncogenic miRNAs or utilizing mimics to reinstate oncogenic miRNA activity. Moreover, the integration of miRNA therapies with traditional treatments can enhance efficacy and mitigate drug resistance. The following summarizes specific therapy techniques and notable research advancements.

### 9.1. miRNA Antagonists (Antagomirs, Anti-miRs, Blockmirs)

Chemically modified oligonucleotides (e.g., antagomirs, locked nucleic acid anti-miRs) with complementary sequences are engineered to target oncogenic miRNAs that are aberrantly expressed in cancer stem cells and possess oncogenic or stem cell-maintaining characteristics [132,133]. These antagonists specifically bind to and obstruct the function of the target miRNA, thereby preventing it from silencing the target gene. For example, treatment of colorectal cancer utilizing cord blood mesenchymal stem cells (cbMSCs)-derived exosomes containing anti-miRNA-221 (anti-miR-221) dramatically decreased tumor proliferation and clone formation and displayed effective anti-tumor effects in in vivo models [134].

### 9.2. miRNA Mimics and Replacement Therapy

We produce double-stranded miRNA mimics that target oncogenic or differentiation-promoting miRNAs, which are either absent or downregulated in cancer stem cells (CSCs). Upon cellular introduction, the mimics can substitute for absent miRNAs and suppress oncogenic target genes [135]. The liposome-encapsulated delivery of synthetic miR-34a reinstated its multi-targeted oncogenic function and suppressed carcinogenesis and progression; however, its clinical development was halted due to immunotoxicity, thereby confirming the pharmacodynamic viability of miRNA treatment [136]. Engineered bacterial microcells (EDVs) administering miR-16 mimics aimed at EGFR-positive tumors decreased PD-L1 expression and mitigated the immunosuppressive microenvironment [137]. Exosomes from adipose tissue-derived mesenchymal stem cells (AMSCs) carrying miR-199a-3p markedly increased the susceptibility of hepatocellular carcinoma (HCC) cells to chemotherapy [138]. Exosomes produced from bone marrow mesenchymal stem cells (BM-MSCs) conveying miR-124 significantly suppressed the proliferation, epithelial–mesenchymal transition (EMT), invasion, and migration of pancreatic cancer cells [139].

### 9.3. Integration of miRNA Therapy with Traditional Treatment Modalities

Integrating miRNA-targeted therapeutics (antagonists or mimetics) with chemotherapy, radiation, targeted therapy, or immunotherapy can enhance efficacy and mitigate resistance mechanisms associated with traditional treatments. Radiotherapy facilitates the selective encapsulation and release of miR-603 from exosomes, alleviating its suppression of IGF1/IGF1R and MGMT, hence resulting in resistance. The co-administration of exogenous miR-603 inhibited the radiotherapy-induced increase of cancer stem cell transformation and DNA repair, resulting in a substantial synergistic anti-tumor impact when combined with radiotherapy and temozolomide (IR/TMZ) [140]. Sub-lethal doses of chemotherapy induced breast cancer cells to release exosomes (EVs) enriched with miR-9-5p, miR-195-5p, and miR-203a-3p, which facilitated cancer stem cell amplification by suppressing ONECUT2. Targeting the EV-miRNA-ONECUT2 axis concurrently with chemotherapy (e.g., knocking down critical genes for exosome secretion, restoring ONECUT2 function, or blocking certain miRNAs) decreases the likelihood of treatment resistance and enhances the rate of pathological complete remission (pCR) [141].

In conclusion, targeted miRNA therapeutic approaches exhibit distinct benefits: by specifically modulating oncogenic or oncostatic miRNAs, they can accurately address the fundamental traits of cancer stem cells (e.g., self-renewal, differentiation inhibition, and drug resistance), offering a novel perspective to combat tumor recurrence and metastasis. The advantage resides not only in the direct elimination of tumor cells or the induction of differentiation but also in the capacity to efficiently reverse immunosuppression within the tumor microenvironment, enhance the efficacy of traditional therapies, and surmount drug resistance. The use of innovative delivery mechanisms, such as exosomes produced from stem cells, is anticipated to enhance targeting and safety. Therefore, the in-depth development of miRNA-based targeted therapies, especially the optimum combination with standard therapies, has a broad clinical application promise and is an important development direction for future tumor therapy.

Furthermore, investigating the organ-specific processes of miRNAs in small-cell-carcinoma stem cells (CSCs) is crucial for formulating more precise and successful treatment options that address the distinct aspects of the tumor microenvironment in various organs. The pore architecture of hepatic sinusoidal endothelial cells in the liver facilitates the penetration of big substances, potentially affecting CSC behavior. Furthermore, cancer stem cells (CSCs) facilitate metastasis via metabolic reprogramming: CSCs exhibiting increased glycolytic activity are predisposed to metastasize to the lungs or brain, whereas those with augmented oxidative phosphorylation (OXPHOS) are inclined to metastasize to the liver [142]. In the Huh7 cell line, double-labeled CD133+EpCAM+ cells demonstrate the greatest tumor-initiating capacity, need only 500 of these cells to successfully generate tumors [143,144]. In breast cancer, CD44^+^CD24^−^/low serves as a characteristic marker, with merely 100 of these cells necessary to initiate carcinogenesis [5]. By comprehending these organ-specific mechanisms, we can formulate tailored miRNA therapeutics that specifically engage distinct signaling pathways and genes within certain organs, so markedly improving therapy efficacy while minimizing off-target consequences and enhancing patient outcomes.

## 10. Conclusions and Future Perspective

This paper examines the regulatory function of miRNAs in CSCs and their prospective use as biomarkers. The biological characteristics of CSCs, as a critical element in tumor recurrence and drug resistance, are meticulously governed by various signaling pathways. In recent years, the regulatory function of miRNAs in CSCs has increasingly attracted attention. Research indicates that miRNAs can profoundly influence the characteristics of CSCs, including self-renewal, apoptosis, invasiveness, proliferation, and metastasis, by targeting essential genes or signaling pathways. Consequently, targeting miRNAs is anticipated to be a fundamental approach for eradicating CSCs and preventing tumor recurrence, drug resistance, and metastasis. These findings establish a novel theoretical foundation for the formulation of miRNA-based cancer therapeutic strategies. Table 1, Table 2, Table 3, Table 4 and Table 5 and Figure 1 and Figure 2 demonstrate the relevant molecular mechanisms by which different miRNAs regulate CSC chemoresistance, radiotherapy resistance, self-renewal, migration, invasion, apoptosis, proliferation, cell cycle and as biomarkers.

Despite extensive research on the regulatory function of miRNAs in CSCs, numerous questions persist unanswered. The precise mechanisms of miRNA action across various cancer types remain inadequately elucidated, as distinct miRNAs may exhibit dual functions in different tumors. This necessitates further investigation into their roles within specific tumor microenvironments and a comprehensive analysis of the regulatory networks of miRNAs in CSCs to formulate miRNA-based therapeutic strategies. The clinical utilization of miRNAs as biomarkers continues to encounter obstacles, such as the establishment of standardized assays and the validation through multicenter clinical trials. Moreover, miRNA-based therapeutic approaches must confront challenges including clinical translation, targeted delivery mechanisms, and safety concerns.

It is anticipated that enhancing multidisciplinary collaboration will facilitate the prompt realization of clinical translation. Through these researches, we hope to promote miRNAs to move from mechanism research to individualized precision therapy, and to provide new tools and strategies for the precision treatment of cancer.

## Figures and Tables

**Table 1:** Role of miRNAs in cancer chemotherapy and radiation sensitivity modulation.

Events	miRNA	Cancers	Cell Lines	Levels	Target	Mechanism	Function	Reference
Radiochemo-therapy sensitivity	miR-378a-3p/ miR-378d	BC	CAL51, MDA-MB-231, MCF-7	↑	DKK3, NUMB	Suppression of DKK3 and NUMB expression activates the WNT/β-catenin and Notch signaling pathways.	Improved characterization of BCSCs resulting in chemotherapy resistance	[21]
	miR-1275	BC	SUM-1315, MDA-MB-231, MCF-7, MCF-7/ADR	↓	MDK	Upregulation of MDK and activation of the PI3K/AKT signaling pathway	Enhanced characterization of BCSCs, thereby promoting chemoresistance in breast cancer	[22]
	miR-508-5p	EC	Ishikawa	↓	SOX2	Promotes SOX2 expression	Promoting self-renewal and chemotherapy resistance in ECSCs	[23]
	miR-181a	HGSOC	HEYA8, OV81.2-CP10, OCI-P5X, OVCAR4	↑	SFRP4	Suppresses SFRP4 expression, thereby facilitating the activation of the Wnt/β-catenin signaling pathway	Facilitating self-renewal and chemoresistance in OCSCs	[24]
	miR-485-5p	OSCC	OC3, OC3IV, CGHNC9 (C9), C9IV3, HSC3, OECM1	↓	KRT17	Promotion of KRT17 expression	Promoting self-renewal and enhancing chemotherapy resistance in OCSCs	[25]
	miR-148a	CRC	T84, SW620, HT29, LS174T, HCT116, CPP1, CPP6, CPP14, CPP24, CPP25, CPP35 CPP19, CPP30, CPP36, CTC44	↓	PXR	Enhanced PXR expression promotes the expression of ALDH1A1, ABCG2 and CYP3A4.	Promoting self-renewal of cancer stem cells, chemotherapy resistance, and thus increased tumor recurrence	[26]
	miR-186	HCC	Huh7, Hep3B	↓	PTPN11	Upregulation of PTPN11 expression	Enhanced self-renewal, tumor-forming capacity, and chemotherapy resistance of LCSCs	[27]
	miR-206	NSCLC	PC-9, HCC827	↓	c-Met	Promotes c-Met expression and activates its downstream Akt and Erk signaling pathways	Enhancement of HGF-induced resistance to gefitinib in gefitinib-resistant lung cancer cells	[28]
		HCC	Huh7, HepG2, Hep3B, CSQT-2, PLC, HCCLM3	↓	EGFR	Promotion of EGFR expression	Promoting self-renewal and proliferation of LCSCs while decreasing drug sensitivity of hepatocellular carcinoma cells to sorafenib and cisplatin	[29]
		iCCA	HiBEC, HUCCT1, RBE, HUVECs, 293T, NFs, CAFs	↓	LASP1, Anxa2	Promoting the expression of LASP1 and Anxa2 increases Nanog expression and promotes CAF formation.	Enhancement of stemness characteristics, proliferation, migration and invasion of cholangiocarcinoma cells while reducing sensitivity to gemcitabine	[30]
	miR-34a	CRC	RKO, DLD-1, SW480, HCT116, HEK 293 T	↓	LRPPRC, MDR1	Promotes LRPPRC expression and maintains MDR1 stability	Reduces the sensitivity of colorectal cancer cells to the chemotherapeutic drug 5-FU	[31]
	miR-497	PC	BxPC-3, AsPC-1	↓	NFκB1	Promotion of NFκB1 expression	Promote chemoresistance, migration and invasiveness of PCSC	[32]
	miR-379-5p	OC	OVCAR3, SKOV3, OV2008	↓	RAD18	Promotion of the RAD18/Polη axis	Promoting self-renewal and chemoresistance in OCSCs	[33]
	miR-7-5p	CRC	HCT116, RKO	↓	KLF4	Promotion of KLF4 expression	Enhancement of CRCSC self-renewal and resistance to radiotherapy	[34]
	miR-29b-3p	BC/LC	A549, MCF7, LLC1	↓	DNMT3B, PIK3R1, AKT2, Bcl-2, RBL1	Promotion of DNMT3B, PIK3R1, AKT2 and Bcl-2 expression and promotion of RBL1 expression	Enhancement of CSC sensitivity to radiotherapy and inhibition of their self-renewal and invasive capacity	[35]
	miR-339-5p	ESCC	KYSE30, KYSE180, KYSE30R, KYSE180R	↓	USP8	Promotion of USP8 expression	Promoting stem cell self-renewal and radiotherapy resistance, thereby reducing ESCC sensitivity to radiotherapy	[36]
	miR-449a	PCa	LNCaP, PC-3, DU-145	↓	c-Myc	Promotion of c-Myc expression Further inhibition of Cdc25A expression	Attenuates PCa cell sensitivity to ionizing radiation and inhibits G2/M phase blocking	[37]
	miR-495	NPC	5-8F, 5-8F-IR	↓	GRP78	Promotion of GRP78 expression	Inhibition of nasopharyngeal carcinoma cell sensitivity to radiotherapy and promotion of the EMT process	[38]

**Table 2:** Regulation of CSC self-renewal by miRNAs.

Events	miRNA	Cancers	Cell Lines	Levels	Target	Mechanism	Function	Reference
Self-renewal	miR-2117	HCC	Huh7, HCCLM3, HepG2	↓	SOX2	Promotion of SOX2 expression	Promote the self-renewal and tumorigenic potential of LCSCs while reducing the sensitivity of HCC cells to chemotherapeutic agents	[44]
	miR-194	HCC	HUH7, HCCLM3	↓	RAC1	Promotion of RAC1 expression	Enhancement of the self-renewal and tumorigenic potential of LCSCs while decreasing the sensitivity of HCC cells to sorafenib	[45]
		EC	Eca-109, TE-13	↓	PRC1, Wnt/β-catenin	Promotion of PRC1 expression	Promotes ECSC proliferation, invasion and self-renewal while reducing apoptosis	[46]
	miR-342-3p	HCC	SMMC-7721	↓	HDAC7, PTEN	Enhancement of HDAC7 expression and inhibition of PTEN expression	Promoting self-renewal of LCSCs	[47]
	miR-130b-5p	CC	HeLa	↓	ELK1	Promotion of ELK1 expression	Promoting self-renewal, proliferation, migration and invasion of CCSCs while inhibiting apoptosis	[48]
	miR-203	GBM	CD133+GBM-SCs	↓	—	Promote the expression of stem cell-related genes	Promoting self-renewal, proliferation and stemness characteristics of GSCs while reducing apoptosis	[49]
		CRC	HCT-116, HT-29	↓	GATA6	Promotion of GATA6 expression	Promote self-renewal and migration of CRCSCs and enhance the expression of stem cell markers such as CD44	[50]
		AML	KG-1a, MOLM13	↓	Survivin, Bmi-1	Promotion of Survivin and Bmi-1 expression	Promoting proliferation and self-renewal of LSCs	[51]
	miR-630	PC	PANC-1, HPDE, HEK-293T	↓	PRKCI	Promotion of PRKCI expression	Enhancing self-renewal and tumor formation in PCSC	[52]
	miR-486-5p	NSCLC	H460, A549, H1299, LT73, HEK-293	↓	PIK3R1	Promotes the expression of p85, which promotes the activation of the PI3K/Akt pathway	Enhancing self-renewal and tumor initiation of cancer stem cells (CD133+)	[53]
		GBM	GBM1A, GBM1B, GBM-KK, Mayo39, A172, HEK293FT, 293T	↑	PTEN, FoxO1	Inhibition of PTEN and FoxO1 expression	Promoting self-renewal and survival of GSCs and enhancing their tolerance to ionizing radiation.	[54]
		HCC	Huh7, Hep3B, Li-7, PLC, 97H, 97L, LM3, HepG2	↓	Sirt1	Promotion of Sirt1 expression	Promoting self-renewal and tumor-forming capacity of LCSCs	[55]
	miR-122-5p	CC	CaSki, CD133+CaSki	↓	SOX2	Promotion of SOX2 expression	Promoting self-renewal and differentiation of CCSCs	[56]
	miR-182	AML	MOLM-13, THP1, K562, OCI-AML3	↓	BCL2, HOXA9	Promotion of BCL2 and HOXA9 expression	Promoting LSC self-renewal and acute myeloid leukemia	[57]

**Table 3:** Regulation of CSC migration ability and invasiveness by miRNAs.

Events	miRNA	Cancers	Cell Lines	Levels	Target	Mechanism	Function	Reference
Migration	miR-101	HCC	HepG2, SUN387, SNU398, SNU423, SNU449, Huh7, SK-HEP-1	↓	ANXA2	Enhances ANXA2 expression and activates ERK signaling pathway	Promoting self-renewal, proliferation and migration of LCSCs	[71]
	miR-7	BC	MDA-MB-231 (MB231), 231BoM-1833, 231BrM-2a, CN34, CN34-BoM2d, CN34-BrM2c, MCF7, MCF7-BoM2d, 293TN	↓	KLF4	Promotion of KLF4 expression	Enhancing self-renewal and brain metastasis of BCSCs	[72]
		BC	MCF-7, SK-BR-3, MDA-MB-231, LD	↓	RELA	Promotion of RELA and ESAM expression	Promoting the transfer capacity of BCSCs	[73]
	miR-148/152 family	GC	MKN45, AGS, KATO-III, NCI-N87, SNU-1, GES-1	↓	ITGA5	Promotion of ITGA5 expression	Promoting self-renewal and tumor-forming capacity of GCSCs	[74]
	miR-139-5p	CRC	HCT116	↓	E2-2	Promotes E2-2 expression and activates the Wnt/β-catenin/TCF7L2 signaling pathway	Promoting self-renewal and transfer of CCSCs	[75]
		IH	HemSCs	↓	IGF-1R	Promotion of IGF-1R expression	Promoting proliferation, migration, self-renewal and differentiation of HemSCs	[76]
	miR-145-5p	HCC	HEK-293T	↓	COL4A3	Promotion of COL4A3 expression	Promoting self-renewal, migration and invasion of LCSCs	[77]
	miR-150-3p	CRC	HCT116, NCM460, CD133+CD44+HCT116	↓	SLCO4A1	Promotion of SLCO4A1 expression	Promote migration, invasion, sphere formation and tumorigenicity of CCSCs	[78]
Invasiveness	miR-128-3p	BC	MCF-7, ZR-75-1, T47D, MB231, MCF-10A	↓	NEK2	Promotion of NEK2 expression	Promote BCSC proliferation, migration, invasion, and self-renewal	[79]
		GBM	GSC103, GSC107, GSC108, GSC109, GSC111, GSC112	↓	LBH	Promotes TNF-α expression by targeting LBH, which in turn promotes the activation of the NF-κB signaling pathway	Promote GSC proliferation, invasion and self-renewal	[80]
	miR-9-5p	PCa	PC3, DU145	↑	NUMB	Inhibition of NUMB expression	Promoting proliferation, migration, invasion and self-renewal of PCSCs	[81]
	miR-210	CRC	HT29, HCT15, Colo205, SW1116, HEK293	↑	STMN1	Inhibition of STMN1 expression	Promoting invasiveness of CRCSCs	[82]
	miR-27b-5p	OC	SKOV3, CAOV3, HO8910, A2780, IOSE80	↓	SIRT5	Promotion of SIRT5 expression	Promote migration, invasion and self-renewal of OCSCs	[83]
	miR-17-5p	GC	SGC-7901, MGC-803, AGS, 293T	↑	MKL-1	Inhibition of MKL-1 expression	Promoting self-renewal and invasiveness of GCSCs	[84]
	miR-7-5p	GC	BGC-823, SGC-7901	↓	Smo, Hes1	Promotion of Smo and Hes1 expression	Promoting the invasive capacity of GCSCs	[85]
	miR-196a-5p	GC	SNU-5, BGC-823	↑	Smad4	Inhibition of Smad4 expression	Promoting self-renewal and invasiveness of GSCs	[86]
		GBM	U87, U251, HEK 293T	↑	FOXO1	Inhibition of FOXO1 expression	Promotion of GSC proliferation, migration and invasion and inhibition of apoptosis	[87]

**Table 4:** Role of miRNAs in the regulation of apoptosis, proliferation, and cell cycle of CSCs and as biomarkers.

Events	miRNA	Cancers	Cell Lines	Levels	Target	Mechanism	Function	Reference
Apoptosis and proliferation	miR-145-5p	GBM	U87, SHG44, 293T, pGSCs	↓	TCTP	Promotion of TCTP expression	Promotes GSC proliferation and reduces apoptosis	[91]
	miR-146b-3p	PC	Panc-1, SW1990, ASPC-1, PC-3, Mia-paca-2, BxPC-3	↓	MAP3K10	Promotion of MAP3K10 expression	Promotes proliferation and self-renewal of P-CSCs and reduces apoptosis	[92]
	Conjunction of miR-124, miR-128, and miR-137	GBM	U251, U343, BE(2)C, Kelly, 3565, 3128, 1123NS, 1919, 19NS, 84NS	↓	SP1, MYB, TCF12, TCF3, EGFR, SRC, CDC42	Promotes the expression of relevant genes	Promotes proliferation, survival and self-renewal of GSCs	[93]
	miR-21	OSCC	SCC-25	↑	CTNNB1, CCND1, BAX, BCL-2, CASP3	Promotion of OCT4, SOX2, NANOG, BCL-2 and CCND1 expression and inhibition of BAX and CASP3 expression	Promoting self-renewal, migration and invasion of OCSCs	[94]
	miR-873	PC	hTERT-HPNE, PANC-1, SW1990, MIA PaCa-2	↓	PLEK2	Promotes PLEK2 expression and further activates PI3K/AKT pathway	Promotes self-renewal and proliferation of PCSC and inhibit apoptosis of cancer cells	[95]
	miR-21-5p	PC	PANC-1, AsPC-1, PC-3, Capan-1, HPC-Y5, THP-1	↑	KLF3	Inhibition of KLF3 expression	Promotes differentiation, self-renewal capacity, invasion and migration of PCSCs, and inhibits apoptosis.	[96]
	miR-202-5p	PC	HPDE6-C7, PANC-1, BxPC-3, MIAPaCa-2	↓	ANP32E	Promotion of ANP32E expression	Promotes proliferation and self-renewal of PCSCs and inhibit apoptosis of cancer cells	[97]
Cell cycle	miR-338-5p	GC	MKN45, MKN74, GES-1	↓	BAK, BIM	Promotes the expression of BAK and BIM	Suppresses the transition of GCSCs from G0/G1 to S and G2/M phases	[98]
	miR-302a/d	HCC	HepG2, Huh7	↓	E2F7	Promotes the expression of E2F7	Decrease in the proportion of G1 phase cells and increase in the proportion of S and G2/M phase cells, accelerating cell cycle progression	[99]
	miR-449b	CRC	SW1116	↓	CCND1, E2F3	Promotion of CCND1 and E2F3 expression	Promotes cell transition from G1 to S phase, accelerates cell cycle progression and enhances cell proliferation	[100]
	miR-92a-3p	CC	Ect1/E6E7, CaSki, SiHa, HeLa, ME180, MS751, C-33A	↑	LATS1	Inhibition of LATS1 expression	Promotes G1/S transition and accelerates cell cycle progression	[101]
Biomarker	miR-637	ESCC	KYSE70, KYSE450	↓	WASH	Enhanced expression of WASH, which promotes the production of IL-8	Promoting CSC self-renewal and tumor growth and as a potential prognostic marker for esophageal cancer	[102]
	miR-638	HCC	HuH-7-Luc, HUVECs	↑	VE-cadherin, ZO-1	Inhibits the expression of VE-cadherin and ZO-1	Disrupts the tight junctions between endothelial cells and increases vascular permeability, thereby promoting intrahepatic metastasis of hepatocellular carcinoma cells	[103]
	miR-1260b	BC	MCF-10A, MCF-7, BT-474, SKBR-3, MDA-MB-231	↑	CASP8	Inhibition of CASP8 expression	Inhibits apoptosis and promotes proliferation, migration and invasion of breast cancer cells	[104]
	miR-221/222	GBM	—	↑	p27, p57, PTPµ, TIMP3, Akt, SOCS3, PUMA, Cx43	Inhibits expression of p27, p57, PTPµ, TIMP3, PUMA, Cx43, etc. Promotes the expression of Akt, SOCS3, etc.	Promotion of glioblastoma proliferation, migration, invasion, radiotherapy resistance	[105]
	miR-23a-3p	HGSOC	OSPC2, OVCAR3	↑	APAF1	Inhibition of APAF1 expression	Inhibition of tumor cell apoptosis, thereby promoting a platinum drug-resistant phenotype	[106]

**Table 5:** Regulatory roles of multiple key miRNAs in various CSCs.

miRNA	Cancers	Cell Lines	Levels	Target	Mechanism	Function	Reference
miR-34a	BC	MDA-MB-231.SC, MCF-7.SC	↓	C22ORF28	Promotes the expression of C22ORF28	Promotes BCSC self-renewal, proliferation, and apoptosis inhibition	[120]
	BC	MCF7, MCF7/ADR, HEK293T		NOTCH1	Promotes the expression of NOTCH1	Promotes chemotherapy resistance in BCSC and inhibit apoptosis	[121]
	PC	-		MET	p53/miR-34-MET signaling axis synergistically regulates	Activates PCSC self-renewal, migration, and invasion	[122]
	PC	LNCaP, PC3, DU145, VCaP, RWPE-1		CD44, Cyclin D1, c-Myc, BCL-2	Promotes the expression of relevant target genes	Promotes PCSC self-renewal, proliferation, and suppression of apoptosis	[123]
	CRC	CCSC1/2/3/4/5	↓	Notch1	miR-34a binds to Notch1 mRNA through mutual sequestration, forming a threshold response that converts continuous Notch signals into a bimodal (high/low) output.	Adjusting the asymmetric division of CCSCs promotes tumor growth and maintains differentiation balance.	[124]
miR-137	CC	SW480, hPCCs, T4056		DCLK1	Promotes the expression of DCLK1	Enhancing the malignant phenotype of colon cancer stem cells	[125]
	PC	AsPC-1,PANC-1		KLF12	Promotes KLF12 expression	Enhancing stem cell properties of pancreatic cancer cells	[126]
	GBM	HF2354, HF2355, HF2414, HF2359, HF2485		RTVP-1	Promotes RTVP-1 expression	Promotes GSC self-renewal and inhibit differentiation	[127]
	BC	MCF-7, SKBR-3, MDA-MB-231		FUNDC1	Promotes FUNDC1 expression	Activates mitochondrial autophagy	[128]
miR-21	OSCC	SCC-25	↑	CTNNB1, CCND1, BAX, BCL-2, CASPASE3	Promotion of OCT4, SOX2, NANOG, BCL-2 and CCND1 expression and inhibition of BAX and CASPASE3 expression	Promoting self-renewal, migration and invasion of OCSCs	[94]
	PDAC	Panc-1, MiaPaCa-2, BxPC3		E-cadherin, Vimentin, Snail, Zeb1, Wnt-11	Regulation of the Wnt-11 pathway	Maintains CSC self-renewal, maintain tumor initiation capacity, and increase chemotherapy resistance	[129]
	CC	HCT-116, HT-29, CR-HCT-116, CR-HT-29		PDCD4, TGFβR2	Activates the Ras/RREB1 pathway and inhibit the transcription of the miR-143/145 cluster	Promotes dryness, tumor growth, and chemotherapy resistance	[130]
	BC	MDA-MB-231		PTEN	Inhibits PTEN expression and activate the PI3K/AKT pathway	Enhances the self-renewal, proliferation, migration, and invasion capabilities of BCSC	[131]

## Data Availability

Not applicable.
